# Emerging Hybrid Nanocomposite Photocatalysts for the Degradation of Antibiotics: Insights into Their Designs and Mechanisms

**DOI:** 10.3390/nano11030572

**Published:** 2021-02-25

**Authors:** Karuppannan Rokesh, Mohan Sakar, Trong-On Do

**Affiliations:** 1 Department of Chemical Engineering, Laval University, Quebec, QC G1V 0A8, Canada; rokesh.karuppannan.1@ulaval.ca (K.R.); m.sakar@jainuniversity.ac.in (M.S.); 2 Centre for Nano and Material Sciences, Jain University, Bangalore 562112, Karnataka, India

**Keywords:** photocatalyst, hybrids, nanocomposites, antibiotics, degradation, contaminants of emerging concerns

## Abstract

The raising occurrence of antibiotics in the global water bodies has received the emerging concern due to their potential threats of generating the antibiotic-resistive and genotoxic effects into humans and aquatic species. In this direction, the solar energy assisted photocatalytic technique offers a promising solution to address such emerging concern and paves ways for the complete degradation of antibiotics with the generation of less or non-toxic by-products. Particularly, the designing of hybrid photocatalyticcomposite materials has been found to show higher antibiotics degradation efficiencies. As the hybrid photocatalysts are found as the systems with ideal characteristic properties such as superior structural, surface and interfacial properties, they offer enhanced photoabsorbance, charge-separation, -transfer, redox properties, photostability and easy recovery. In this context, this review study presents an overview on the recent developments in the designing of various hybrid photocatalytic systems and their efficiency towards the degradation of various emerging antibiotic pharmaceutical contaminants in water environments.

## 1. Introduction

**Figure 1:**
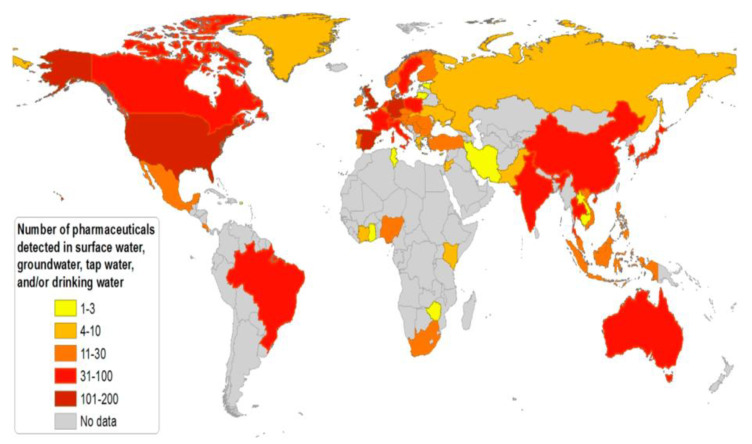
Number of pharmaceutical compounds detected in surface, ground and drinking water systems in worldwide [4].

Pharmaceutical industry is one of the important and largest industries worldwide and at the same time, a large amount of contaminations is being generated by the pharmaceutical products. These pharmaceutical products are largely disbursed at high quantities into the environment by purposely and accidentally. Consequently, these pharmaceutical compounds can be found in different environmental compartments such as soil, water surfaces, and even in drinking water. Especially, these pharmaceutical products are frequently detected in natural and wastewater system [1,2]. The amount of pharmaceutical pollutants and their metabolites collection in water bodies are not high-pitched (ng to mg per liter), however, these pharmaceutical molecules are specifically designed to initiate the biological response at very low concentration levels. Therefore, it may lead to some adverse effects on biological system and human health such as aquatic toxicity, high resistance bacteria, acute and chronic disease, hormonal and endocrine disruption. Moreover, most of the pharmaceutical drugs possess very stable chemical structure and non-biodegradable properties. Therefore, the detection and removal/degradation of pharmaceutical compounds in the water system has been evolved as a growing concern in worldwide (Figure 1), which is essentially due to their potential toxicity and hazardous to the living ecosystems and human beings [3].

Among the various pharmaceutical products, the antibiotics have been received more attention as they generate serious toxicity and produce long-term chronic effects to humans and ecosystems, whereas the antibiotic residues generate serious environmental health issues such as antimicrobial resistivity, antibiotic resistive bacteria and genes modifications. According to the world health organization, the antimicrobial resistance is an emerging problem, which generates multi-drug resistant infections to human and animals [5,6]. Therefore, the antibiotics are considered as “contaminants of emerging concerns” or “emerging pollutants” due to their potential toxicity and their rising occurrence in global water bodies. In addition, the antibiotics residues in the environment could result into various adverse effects and generate stable organic by-products, which are difficult to degrade by the conventional waste-water treatment processes and they could cause the generation of secondary pollutions as well as lead to increase the population of antibiotics resistance bacteria. Therefore, there is an urgent need to address this issue and to develop an efficient technique to remove/destroy these pollutants from water/waste-water [3,7].

The various available techniques to remove and degrade the water/wastewater contaminating pharmaceutical pollutants include adsorption, microbial degradation, photocatalysis, ozonolytic, electrocatalysis and membrane filtration processes [3,8]. Of these techniques, the photocatalysis offers a promising solution for the effective degradation of antibiotics contaminants in water using solar energy [3,9,10,11], where the strong redox reactions of photocatalysis offer effective mineralization, high degradation efficiency, less byproducts and/or simple/non-toxic degradation products. However, the photocatalytic efficiency of photocatalysts mainly depends upon many crucial features such as suitable band edge position, narrow band gap energy, reduced charge recombination, enhanced charge separation, transfer and surface-active sites [10]. Accordingly, considerable efforts have been made to achieve these properties by constructing hybrid nanocomposite structures of photocatalysts with controlled preparation methods [12]. As described, these hybrid nanocomposites fundamentally offer enhanced surface and catalytic properties delivered by large surface area, rich active sites, extended photoabsorbance, higher charge generation, improved interfacial charge separation and strong redox properties [9,11,12,13]. Hence, these hybrid nanocomposite photocatalysts effectively degrade and mineralize the antibiotics in water in presence of solar energy. In this context, this review is mainly focused on the recent developments in the design and synthesis of hybrid nanocomposite photocatalysts and their potential photocatalytic performance towards the degradation of antibiotics in water.

## 2. Photocatalysis: An Overview

**Figure 2:**
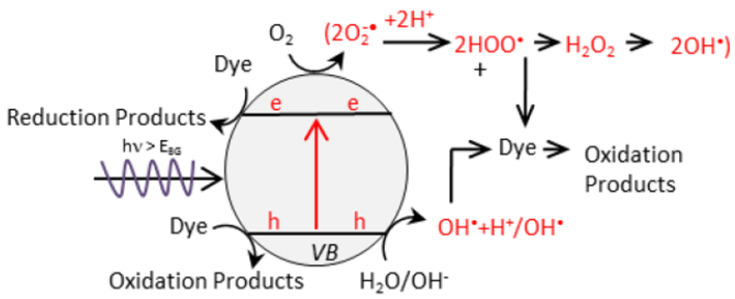
Schematic diagram of photocatalytic reaction mechanism [14].

Photocatalysis is a semiconductor based photoinduced advanced oxidation process, which has received great attention in environmental remediation as it can utilize the solar energy to efficiently degrade the emerging pharmaceutical contaminants into non-toxic by-products. The photocatalytic process undergoes to four main steps; (i) photo-absorption, (ii) charge separation, (iii) charge transfer and (iv) redox reaction. A semiconductor photocatalyst has the valence band (VB) and conduction band (CB) that separated by an energy gap known as band gap energy (Eg). Initially, the semiconductor photocatalyst undergoes the photo-absorption that excites the electrons from VB to CB and leaving the holes in VB. This charge separation further leads to the promotion of electrons to the photocatalyst surface to perform the reduction reactions and the holes to perform the direct oxidation process as depicted in Figure 2 [14,15,16].

It should be noted that both the oxidation and reduction reactions are fundamental processes in photocatalysis, which essentially lead to the primary and secondary degradation processes. The primary reaction involves the direct hole oxidization established by the photogenerated holes (h^+^), while the secondary reaction involves the reaction of reactive free radicals such as hydroxyl (^•^OH) and super oxide radicals (O_2_^•−^), which are formed by the water oxidation by holes and reduction of oxygen molecule by electrons, respectively. Thereby, these radicals further involve in the reduction and oxidation reaction over the pollutants and degraded them completely [17].

The photo-redox active semiconductor materials play key roles in photocatalytic process due to their superior crystallinity, band structure, surface properties [18]. The higher order of crystallinity and crystal defects tend to reduce the probability of recombination of photo-generated electron-hole pairsand offer efficient charge transfer in the system. The semiconductor photocatalysts with suitable band gap and band alignment offer an efficient photoabsorbance and potential charge diffusion, and their large surface area offer higher colloid dispersion and surface-active sites, which facilitate the enhanced adsorption of reactant molecules and higher photocatalytic activities [10,19,20]. However, the single component photocatalytic materials possess several limitations such as wide band gap energy, high numberof structural defects, reduced photo-generated charge carriers separation, greater charge recombination and less charge transfer. Further, the photo-corrosion properties of the semiconductor photocatalysts could limit the overall activity and stability of the system [21,22].

## 3. Hybrid Photocatalysts: An Overview

**Figure 3:**
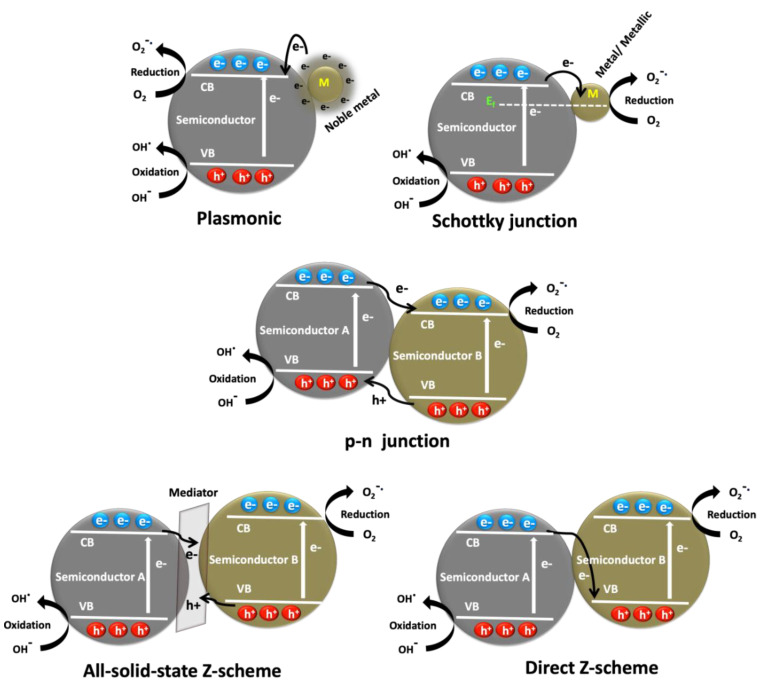
Schematic illustration of hybrid photocatalyst with different photocatalytic mechanism.

Nanomaterials offer greater efficiencies towards photocatalytic degradation due to their high specific surface area, surface reactive sites and the surface-dependent photocatalytic properties. Nevertheless, the nanostructured photocatalysts also show several drawbacks such as limited solar light absorption, poor charge separation, slow charge transfer, higher charge recombination and less stability [23,24]. Hence, to overcome these issues, the construction of hybrid nanocomposite photocatalysts was largely adopted and the construction of hybrid heterostructure nanocomposites can be done by coupling of two or more nanoscale materials with specific properties [25,26]. For instance, the design of such hybrid nanocomposite systems with different photocatalytic mechanisms such as Schottky, plasmonic, Z-scheme and p-n heterojunction, etc., are illustrated in Figure 3. These hybrid systems essentially provide efficient surface and interface contacts through their unique mechanisms, thereby improved charge separation and faster charge migration in the system. Moreover, these heterostructures deliver higher surface area and multiple optical properties thereby enhanced photoabsorbance. Besides, the hybrid composites also demonstrate enhanced photo-stability and negligible photo-corrosive properties [22,23,24,25,26,27,28,29,30]. Hence, the designing and application of hybrid nanocomposite photocatalysts seem to be an interesting strategy to improve photocatalytic antibiotic degradation. As a result, there have been many such systems developed and investigated for the antibiotics degradations, which have been discussed in this review. It is known that the hybrid systems are generally consisting of a host material and one or more integrating materials. Accordingly, in this review report, these hybrid systems have been classified based on the key element present in the host material, which essentially governs the design and mechanism of the hybrid photocatalysts.

### 3.1. Bismuth Hybrids

The heterostructure composite composed of uniformly distributed AgI nanoparticles on Bi_12_O_17_Cl_l2_ nanolayer was developed for the effective degradation of sulfamethazine under visible light (300 W Xe lamp). The obtained photocatalytic results showed that the degradation rate of AgI/Bi_12_O_17_Cl_l2_ heterostructure was 7.8 and 35.2 folds higher than that of the pristine Bi_12_O_17_Cl_l2_ and BiOCl, respectively. The achieved remarkable photocatalytic enhancement was mainly attributed to the effective charge transfer occurred via the established interfacial contacts between AgI and Bi_12_O_17_Cl_l2_. In addition, it was observed that the amount of AgI in the AgI/Bi_12_O_17_Cl_l2_ composites played an important role on the charge carriers separation, redox ability and as well as photostability of the system, and the optimized ratio was obtained at 25% [31]. Similarly, the AgI/Bi_2_Sn_2_O_7_ Z-scheme nanojunction hybrid displayed the higher photocatalytic performance towards the tetracycline removal, where it degraded around 83.0% of tetracycline within 50 min under visible light using a 300 W Xe lamp. The nanojunction formed between AgI and Bi_2_Sn_2_O_7_ facilitated a faster interfacial charge transfer and the enhanced photocatalytic performance. Analysis of three-dimensional excitation-emission matrix fluorescence spectra (3D EEMs) and liquid chromatography-mass spectrometry (LC-MS) measurements revealed that the chemical structure of tetracycline was effectively destroyed, and intermediates were generated progressively [32]. A novel direct Z-scheme Bi_7_O_9_I_3_/g-C_3_N_4_ (BCN) heterostructure photocatalyst was successfully constructed by in-situ growth of Bi_7_O_9_I_3_ nanoflowers on ultrathin g-C_3_N_4_nanosheets. The as-developed Bi_7_O_9_I_3_/g-C_3_N_4_ (BCN-0.2) heterojunction photocatalyst was able to easily excited in visible light and showed an excellent photocatalytic activity on doxycycline hydrochloride degradation (~80% in 120 min) and mineralization (~67.8% in 120 min). Moreover, the Z-scheme heterostructure established a stable crystal structure which offered better stability to the composite and also the optimal band arrangement provided efficient photoinduced electrons-hole separation. The radical experiments determined that the hydroxyl and superoxide radicals played predominant role in doxycycline hydrochloride degradation [33]. Interestingly, the in-situ growth of Z-scheme BiOBr/Ag_3_PO_4_ heterojunctions on carbon fiber (CF) cloth was developed by solvothermal-chemical deposition process. The prepared CF/BiOBr/Ag_3_PO_4_ cloth displayed an excellent photocatalytic performance of around 90% tetracycline hydrochloride degradation in 30 min under visible light (300 W xenon lamp with a cut-off filter λ > 400 nm). The higher photocatalytic activity was reached by the decoration of Ag_3_PO_4_, which broadened the photo-absorption range and also enhanced the electron-hole pair separation and migration efficiency. Moreover, the carbon fiber cloth as a flexible porous substrate was used as a filter-membrane to construct multiple photocatalytic setups for degrading antibiotic from flowing wastewater. Moreover, this ternary CF/BiOBr/Ag_3_PO_4_ photocatalyst can be explored as a floating and easily recyclable photocatalyst for eliminating antibiotics in water [34]. 

**Figure 4:**
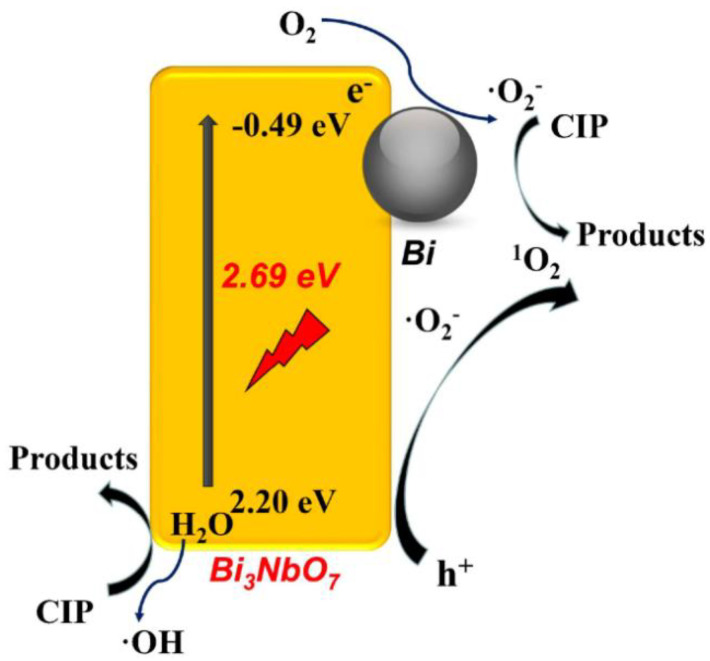
Photocatalytic mechanism for Bi/Bi_3_NbO_7_ composite under visible light irradiation [35].

The two-dimensional (2D) materials have opened up new possibilities in photocatalytic degradation of antibiotics. For example, 2D semiconductor composite with molecular oxygen activation offers unique visible light response and efficient charge carrier separation. Representatively, the fabrication of 0D/2D system composed of 2–5 nm Bi nanodot/Bi_3_NbO_7_ nanosheet semimetal/semiconductor heterojunction composites with enhanced molecular oxygen activation demonstrated excellent degradation efficiency over ciprofloxacin under visible light irradiation (300 W Xe lamp). The formation of strong covalent interaction between the Bi ions (of Bi nanodots) and the Bi-O layer (of Bi_3_NbO_7_ nanosheets) was found to be responsible for the accelerated charge separation and transfer of charges carriers at the interface of the system and boosted up the molecular oxygen activation (Figure 4). As a result, the promoted activation of molecular oxygen into superoxide radicals (O_2_^•−^) and singlet oxygen (^1^O_2_) species significantly improved the photocatalytic degradation efficiency of Bi/Bi_3_NbO_7_ composites, where it was found to be 4.58 folds higher than that of the pristine Bi_3_NbO_7_ [35].

Similarly, the Bi_4_Ti_3_O_12_/I-BiOCl 2D/2D heterojunction composite was constructed via the modified in-situ ion exchange approach at room temperature. The efficient 2D/2D interface and the well-matched band structure provided the efficient inter surfaces contacts, which enhanced the charge transfer rate, while the doped iodine (I^−^) ions significantly improved the visible light absorption in the system. Hence, the as-developed Bi_4_Ti_3_O_12_/I-BiOCl was found to be highly photocatalytic towards the degradation of ciprofloxacin and tetracycline, where it degraded 88.4 and 80.7% of the respective antibiotics [36]. Likewise, the two-dimensional carbon-doped carbon nitride (CCN)/Bi_12_O_17_Cl_l2_ composite demonstrated the outstanding photocatalytic activities for the degradation of tetracycline, which was approximately 2.9, 1.5 and 32.1 folds higher than those of pristine Bi_12_O_17_Cl_l2_, CCN and BiOCl, respectively. The observed improved activity was attributed to the electrostatic interaction between CCN and Bi_12_O_17_Cl_l2_, which offered superior charge transport properties. Furthermore, the obtained 3D excitation-emission spectra (EEMs) indicated that the CCN/Bi_12_O_17_Cl_l2_ composite possessed high mineralization ability for tetracycline degradation [37]. A three-dimensional hierarchical (3D) BiOI/MIL-88B(Fe) metal organic framework (MOF) hybrid nanocomposite was prepared via a simple precipitation method and the composite showed higher solar photocatalytic activity as compared to the pure BiOI towards the degradation of ciprofloxacin. The achieved enhanced catalytic activity was attributed to the formed Z-scheme charge transfer mechanism. In addition, this 3D BiOI/MIL-88B(Fe) (2 wt%) hybrid composite also showed good reusability and long-term stability [38]. More recently, the designing of covalent organic frameworks (COFs)/semiconductor composite received more interest due to their structural flexibility and tremendous catalytic sites. For example, the three-dimensional covalent triazine framework (CTF-3D) was integrated with two-dimensional BiOBr nanoflake to develop the novel BiOBr/CTF-3D composite and investigated for the degradation of tetracycline and ciprofloxacin under visible light irradiation (500 W Xe lamp). The optimum amount of CTF-3D (2%) in the BiOBr/CTF-3D composite was found to be responsible for the observed highest photocatalytic degradation of tetracycline (90.9%) within 50 min and it found to degrade around 60% ciprofloxacin under same condition [39].

### 3.2. Cadmium Hybrids

The development of Z-scheme based heterojunction photocatalytic systems is a promising strategy to degrade antibiotics. The designing of direct Z-scheme CdS/Bi_4_V_2_O_11_ photocatalytic system was found to have higher redox ability than the pure CdS and Bi_4_V_2_O_11_ samples. The CdS/Bi_4_V_2_O_11_ (@3:1) composite showed higher degradation efficiency towards ciprofloxacin (76.97%) and tetracycline (83.70%) removal in 120 min under visible light (λ > 420 nm), also catalyst found enhanced stability for several cycles [40]. Similarly, an in-situ synthesis of CdS/SnO_2_ nanocomposite heterojunctionphotocatalyst displayed 85.4% of tetracycline degradation in 60 min under visible light irradiation (300 W Xe lamp). It was found that the lattice mismatch between CdS and SnO_2_ improved the interfacial contacts and speeded up the charge migration and reduced the electrons and holes recombination rate in the system. Further, the smaller CdS nanoparticles with strong interface interaction significantly enhanced visible photo-absorption and as well as improved the surface charge transfer. In addition, the photo-corrosion of CdS was also potentially prevented by the composite formation with SnO_2_ [41]. The nitrogen-doped carbon supported cadmium sulfide (CdS/NC-T) composites were successfully prepared via a facile in-situ carbonization method using cadmium-based MOF precursors. The as-developed CdS/NC-500 composite found 83% of tetracycline degradation in 60 min under visible light irradiation (300 W Xe-arc lamp), with an apparent rate constant (κ) of 0.0639 min^−1^. The perfect interface contacts between CdS nanoparticles and carbonaceous material improved the photo-generated charge carrier generation and transfer. Moreover, the CdS/NC-500 composite exhibited good stability and reusability during tetracycline degradation [42]. The CdIn_2_S_4_ nano-octahedron and ZnO nanosheet embedded 3D/2D heterostructure composite was found to have a well-established morphology and excellent visible light photocatalytic activity towards the tetracycline hydrochloride degradation (~94% in 40 min). As compared to ZnO and CdIn_2_S_4_ the degradation efficiency of CdIn_2_S_4_/ZnO composite was found to be 1.95 and 4.74 folds higher. The designing of 3D/2D structure offered the effective interface contact and higher photo-generated electron-hole separation, which eventually offered the enhanced photocatalytic performance [43].

### 3.3. Calcium Hybrids

**Figure 5:**
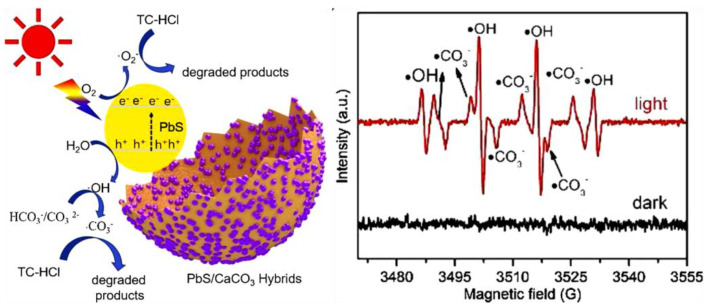
Photocatalytic degradation mechanism and radicals generation of PbS/CaCO_3_ composites [46].

The CaFe_2_O_4_/MgFe_2_O_4_ nanowires-based nanocomposite demonstrated higher photocatalytic degradation of tetracycline (~40%) as compared to their individual counter parts CaFe_2_O_4_ (15%) and MgFe_2_O_4_ (12%) under visible light. In this system, the formed heterostructure in CaFe_2_O_4_/MgFe_2_O_4_ nanowires effectively aligned the positions of their energy bands, thereby prevented the recombination of photo-generated electrons and holes [44]. The 3 wt% CdS QDs decorated p-CaFe_2_O_4_@n-ZnFe_2_O_4_ ternary heterojunctionphotocatalyst was employed to degrade 50 ppm of norfloxacin under visible light irradiation, where it showed 83% of degradation in 90 min, which was around 1.28 times higher than that of CaFe_2_O_4_@ZnFe_2_O_4_. The modified band gap alignment offered the excellent charge transfer characteristics in p-CaFe_2_O_4_@n-ZnFe_2_O_4_ heterojunction composite. In addition, the iso-energetic charge transfer from CdS QDs to CaFe_2_O_4_@ZnFe_2_O_4_ was found to remarkably enhance the photoelectron generation and transfer in the system [45]. In this direction, the carbonate radicals also played significant roles in antibiotics degradation. For example, the carbonate radical (CO_3_^−^) mediated photocatalytic tetracycline hydrochloride degradation was carried out using egg/shell-based PbS/CaCO_3_ composite under solar light irradiation. This composite established a remarkable photocatalytic degradation efficiency of 82% of tetracycline degradation within 40 min (Figure 5) [46].

### 3.4. Cerium Hybrids

The La_2_O_3_/CeVO_4_@halloysite nanotubes ternary composites displayed outstanding photocatalytic activity on tetracycline degradation under visible light irradiation (λ ≥ 420 nm). The optimized composition of La_2_O_3_/CeVO_4_ (0.25:1) at halloysite nanotube composite was found to have a higher photocatalytic degradation of 87.1% in 60 min, which was around 3.89 folds higher than that of CeVO_4_ (22.4% in 60 min). The observed higher photocatalytic activity was attributed to the co-existence of Ce^4+^ and Ce^3+^ pairs in CeVO_4_, which facilitated an enhanced electron-hole pair separation. Further, the La^3+^ ions in La_2_O_3_ were acted as an electron trapper, which enhanced the charge transfer. In addition, the formation of heterojunction between La_2_O_3_ and CeVO_4_ facilitated the higher charge carrier separation and transfer [47]. An in situ loading of Ag_2_O on CeO_2_ spindles was performed to develop the Ag_2_O/CeO_2_ based p-n heterojunction photocatalysts and studied their photocatalytic degradation of enrofloxacin under visible light irradiation (300 W Xe lamp). The obtained results showed that the Ag_2_O/CeO_2_ heterojunction photocatalyst was able to degrade ~87.11% in 120 min and mineralize ~66.82% in 160 min. The mechanism of photo-degradation involved the formation of p-n junction, which established the higher photoinduced charge separation in the system thereby it improved the generation of h^+^ and O_2_^•−^ active species to effectively degrade enrofloxacin [48]. The Ce(MoO_4_)_2_ nanocubes/graphene oxide (CeM/GO) composite demonstrated efficient photocatalytic oxidation and electrochemical reduction for the removal of neurotoxicity antibiotic chloramphenicol. The CeM/GO nanocomposite exhibited an excellent photocatalytic degradation of chloramphenicol (99%) within 50 min under visible light irradiation (500 W tungsten lamp equipped with a UV cutoff filter λ > 400 nm). The superior photocatalytic activity was attributed to the enhanced separation of the photoinduced electrons and holes in CeM/GO nanocomposite [49]. Similarly, a high crystalline pyrochlore Ce_2_Zr_2_O_7_@RGO nanocomposite was prepared by a simple combustion method and studied for the degradation of ciprofloxacin. The nanocomposite found to degrade 89.0% of ciprofloxacin in 60 min under visible light irradiation (250 W Hg light source fitted with a 400 nm cut-off filter). The higher photocatalytic activity of Ce_2_Zr_2_O_7_@RGO nanocomposite was attributed to π-conjugation mechanism of rGO, which prolonged the lifetime of photogenerated electrons by the inhibiting electron-hole recombination. In addition, the presence of oxygen defects was also found to be the reason for the observed improved photocatalytic performance [50].

### 3.5. Cobalt Hybrids

**Figure 6:**
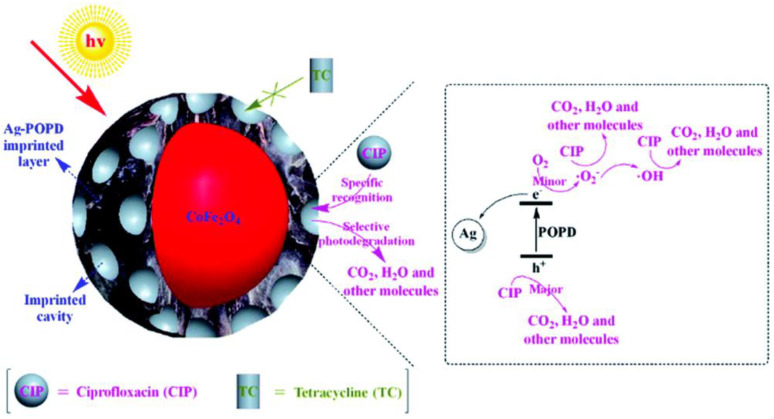
Selective photocatalytic degradation of antibiotics (ciprofloxacin and tetracycline) by imprinted Ag-POPD/CoFe_2_O_4_ [51].

A stable core-shell imprinted silver-(poly-o-phenylenediamine)/CoFe_2_O_4_ spherical core-shell was fabricated by surface imprinting technique and used for ciprofloxacin degradation with good magnetic separation. The introduction of silver-(poly-o-phenylenediamine) (Ag-POPD) into the imprinted layer was found to significantly improve the photocatalytic activity and achieved ~94.38% of ciprofloxacin degradation in 90 min under simulated sunlight (250 W xenon lamp). The imprinted cavities in the imprinted layer offered superior specific recognition capability for the selective degradation of ciprofloxacin (Figure 6). In addition, in this photocatalytic reaction, the holes performed as primary oxidative species and superoxide radicals performed as secondary oxidative species [51].

Similarly, the magnetic ion imprinted heterojunction photocatalyst demonstrated a selective photoreduction of Cu^2+^ and simultaneous photodegradation of tetracycline under visible light. The dual channel ion imprinted POPD-CoFe_2_O_4_ heterojunction photocatalyst was found to selectively reduce the Cu^2+^ due to the abundant presence of Cu^2+^ imprinted cavities in the imprinted layer and therefore the Cu^2+^ rapidly reduced by the electrons in POPD. In addition, this imprinted mesoporous structure offered efficient adsorption of tetracycline molecules followed by the effective degradation on the CoFe_2_O_4_ surface. More importantly, the heterojunction formation between CoFe_2_O_4_ and POPD effectively separated the photogenerated electron-holes, which greatly promoted the photocatalytic selective reduction of Cu^2+^ and as well as photodegradation of tetracycline [52]. The CoFe_2_O_4_@CuS magnetic nanocomposite was studied for the degradation of Penicillin G in aqueous solutions under UV light (18 W UV-C lamp, λ = 253.7 nm). The composite degraded ~70.7% of Penicillin G in 120 min, which was much higher than that of the photolytic degradation of Penicillin G (27.1%) under UV light. Furthermore, this magnetic nanocomposite also showed higher reusability [53]. Further, a magnetic hybrid heterostructure g-C_3_N_4_/Co_0.5_-Zn_0.5_Fe_2_O_4_ photocatalyst was also demonstrated the higher photocatalytic degradation of chloromycetin under visible light irradiation (300 W xenon lamp with UV cut-off filter). This hybrid g-C_3_N_4_/Co_0.5_-Zn_0.5_Fe_2_O_4_ composite showed 2.5 folds higher efficiency than that of pure g-C_3_N_4_. The close interfacial contacts between Co_0.5_-Zn_0.5_Fe_2_O_4_ and g-C_3_N_4_ facilitated an efficient separation of photogenerated electron-hole pairs and improved the photocatalytic activity [54]. The hollow porous Co_2_SnO_4_-SnO_2_/graphite carbon (Co_2_SnO_4_-SnO_2_/GC) nanocube heterojunction designed by calcining the CoSn(OH)_6_ precursors followed by the immersion of carbon coating using a recyclable napkin as graphite carbon source. The as-prepared hollow porous Co_2_SnO_4_-SnO_2_/graphite carbon (Co_2_SnO_4_-SnO_2_/GC) nanocube heterojunction demonstrated a remarkable photocatalytic performance for the degradation of chlortetracycline (83.0% in 80 min) and tetracycline (~80.0% in 80 min) under visible light irradiation (500 W xenon lamp and 420 nm cut-off filter). The observed efficiency was attributed to the synergistic effects among the different multi-junctions, which greatly promoted the separation of the electron-hole pairs and suppressed the charge recombination. In addition, the graphite carbon was found to act as a protective layer, which preserved the activity and stability of Co_2_SnO_4_-SnO_2_/GC heterojunction composites [55].

### 3.6. Copper Hybrids

**Figure 7:**
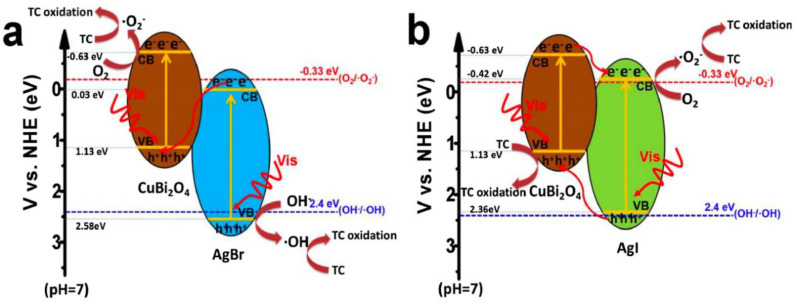
(**a**) Z-scheme mechanism of AgBr/CuBi_2_O_4_ composite and (**b**) type II heterojunction mechanism of AgI/CuBi_2_O_4_ composite [59].

The incorporation of Cu^2+^ into zeolite imidazolate frameworks (ZIF-8) was very beneficial to the formation of hollow porous CuO/ZnO composite and also the excess of Cu^2+^ greatly influenced on the morphology of the composite. Accordingly, the as-developed CuO/ZnO hollow structure composite showed ~87.0% degradation of tetracycline in 60 min with exposure of visible light irradiation (λ > 420 nm), where it was found that the degradation rate was significantly faster than the pure ZnO and CuO. Moreover, the CuO/ZnO hollow structure composite demonstrated excellent reusability and stability [56]. Likewise, the incorporation of CuInS_2_ into Mg(OH)_2_ nanosheets showed the enhanced visible-light photocatalytic activity towards the tetracycline hydrochloride degradation. The effective interface contact between CuInS_2_ and Mg(OH)_2_ improved the charge carriers separation and transfer in the photocatalytic system. Then, the surface roughness of CuInS_2_/Mg(OH)_2_ nanosheets increased the overall adsorption property of the system. Moreover, the photocatalytic activity of CuInS_2_/Mg(OH)_2_ was significantly influenced by the concentration of CuInS_2_, pH value and inorganic ions [57]. A stable and efficient direct Z-scheme CuInS_2_/Bi_2_WO_6_ heterojunction with intimate interface contact was designed over the direct growth of Bi_2_WO_6_ on CuInS_2_ microspheres. The CuInS_2_/Bi_2_WO_6_ heterojunctions found to degrade 90.5% tetracycline hydrochloride via Fenton-aided photocatalytic process and displayed excellent reusability. The direct Z-scheme charge transfer pathway and close interface contact of CuInS_2_/Bi_2_WO_6_ heterojunction were attributed to the remarkable improvement in the separation of photo-generated electrons and holes and leading to the higher photocatalytic activity [58]. The silver halide decorated semiconductors-based composite with visible light activity was developed to solve the problem of antibiotic degradation. In this direction, the AgX (X = Br, I)/CuBi_2_O_4_ heterojunction composites were synthesized via in-situ precipitation method. In comparison with pristine CuBi_2_O_4_, the AgX (X = Br, I)/CuBi_2_O_4_ heterojunction composites showed superior photocatalytic tetracycline degradation performance due to their efficient interfacial charge separation and migration. These two AgX (X = Br, I)/CuBi_2_O_4_ heterojunction photocatalysts displayed distinct photocatalytic performance with diverse photocatalytic mechanisms under visible light irradiation (λ > 420 nm). Accordingly, it was found that the Z-scheme heterojunction was formed between AgBr and CuBi_2_O_4_, which showed the higher reduction potential in CuBi_2_O_4_ and stronger oxidation potential in AgBr, whereas, the type II heterojunction was formed between AgI and CuBi_2_O_4_, which effectively facilitated the separation of electron-hole pairs in the AgI/CuBi_2_O_4_ composite (Figure 7) [59].

Interestingly, a hydrogel composite catalyst based on (HEA/NMMA)-CuS with dual functional properties of adsorption and degradation was studied on the removal of sulfamethoxazole. The adsorption process of sulfamethoxazole on complex hydrogel was well fitted to Langmuir monolayer adsorption and as well as pseudo-second-order rate equation. Accordingly, the hydrogel composite was found to remove ~95.91% and mineralize ~43.56% of sulfamethoxazole under visible light irradiation (500 W xenon lamp, 400 nm glass cut-off filter). The hydroxyl radicals were identified as main reactive species in the degradation process, followed by the intermediates of sulfamethoxazole were identified and possible degradation pathway was also proposed [60]. Likewise, a new approach for the fabrication of p-type CuBi_2_O_4_ and zeoliticimidazolate framework-8 (CBO@ZIF-8) core-shell nanostructure was demonstrated their multifunctional application of both fluoresce detection and photodegradation of antibiotics under visible light irradiation (350 W xenon lamp with a cut-off light filter below 400 nm). The results showed that the CBO@ZIF-8 composite demonstrated an excellent fluorescence-sensing of tetracycline with high sensitivity and selectivity. Correspondingly, it displayed good photocatalytic activity on the degradation of tetracycline in terms of its efficiency, around 75.2% in 120 min and better reusability for five successive cycles [61].

### 3.7. Graphitic Carbon Nitride Hybrids

**Figure 8:**
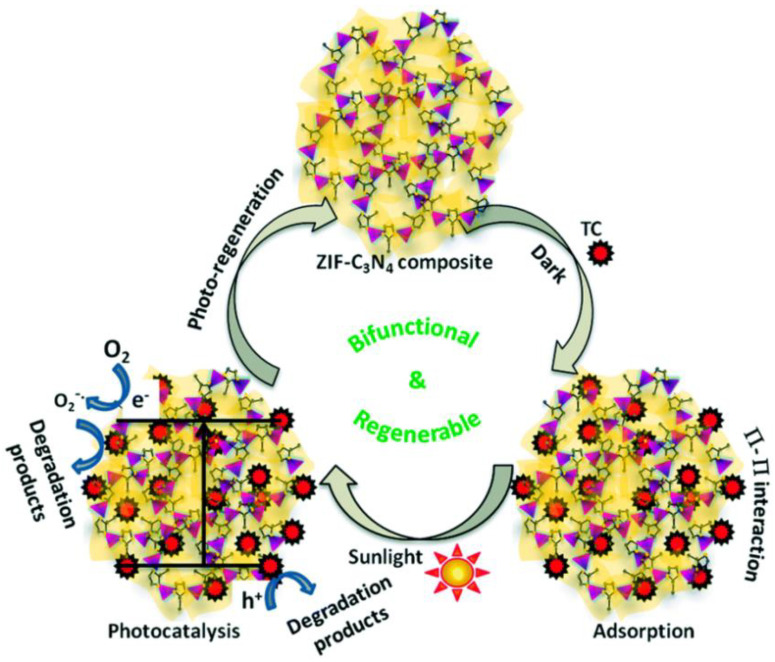
Schematic illustration of adsorption and photocatalytic degradation of tetracycline by C_3_N_4_-ZIF-8 bi-function composite [69].

The ultrathin MoS_2_/graphitic-C_3_N_4_ (MoS_2_/g-C_3_N_4_) hybrid composite was fabricated by establishing well-bonded interface contacts between ultrathin MoS_2_ nanosheets and g-C_3_N_4_. The hybrid MoS_2_/g-C_3_N_4_ with 5 wt% MoS_2_ photocatalyst was found to have higher degradation efficiency for the ciprofloxacin and tetracycline removal, where it exhibited ~81.9 and ~96.0% of ciprofloxacin and tetracycline degradation, respectively in 240 min under visible light source (250 W metal halide lamp with UV cut-off filter). Accordingly, the mechanism was attributed to the accelerated separation and transfer of photogenerated electron-hole pairs in the MoS_2_/g-C_3_N_4_ hybrids mediated by ultrathin MoS_2_ nanosheets. Besides, MoS_2_/g-C_3_N_4_ composite showed excellent recyclability and chemical stability after 10 times reused [62]. The fluorinated graphitic carbon nitride photocatalyst with magnetic properties Fe_3_O_4_/g-C_3_N_4_ (FeGF) was prepared by hydrothermal method and explored for degradation of amoxicillin in water. As compared to bulk g-C_3_N_4_, the fluorinated Fe_3_O_4_/g-C_3_N_4_ composite showed a higher specific surface area (243 m^2^ g^−1^) and easy magnet separation from the medium, which eventually improved the photocatalytic activity in terms of amoxicillin removal and mineralization as well as detoxification of the solution. Fe_3_O_4_/g-C_3_N_4_ exhibited photocatalytic activity under both UV (10 W) and visible lights (500 W), the result showed that the degradation efficiency of amoxicillin under UV light was significantly higher than that observed under visible light irradiation [63]. The graphitic carbon nitride (g-CN) and SmFeO_3_ based Z-scheme g-CN/SmFeO_3_ heterostructure photocatalytic composites were prepared via mixing-ultrasonication process with different weight ratio of g-CN:SmFeO_3_ such as 20:80, 50:50 and 80:20. The as-developed g-CN/SmFeO_3_ (80:20) composite showed potential visible photocatalytic activity on tetracycline hydrochloride degradation. The efficient heterostructure formation and the established better interfacial contact enhanced the light absorption in the entire visible light region and improved the photoinduced charge separation and transfer. Moreover, the heterostructure g-CN/SmFeO_3_ composite demonstrated higher stability and better reusability up to six cycles [64]. Similarly, an easily recyclable T-g-C_3_N_4_/PET composite photocatalyst was fabricated through the dispersion of graphitic carbon nitride (g-C_3_N_4_) into polyethylene terephthalate (PET) nanofibers. The as-prepared T-g-C_3_N_4_/PET nanofibers demonstrated higher photocatalytic stability and reusability for the sulfaquinoxaline and sulfadiazine antibiotics degradation. The significant enhancement in photocatalytic stability and reusability of T-g-C_3_N_4_/PET nanofibers was attributed to the dispersion and recycling functions of the PET. The as-developed composite photocatalyst facilitated the complete degradation of sulfaquinoxaline under solar irradiation (Q-SUN Xe-1 lamp with daylight) within 2.5 h [65]. The metal-free heterojunction photocatalytic system using the hexagonal boron nitride (h-BN)-dispersed-g-C_3_N_4_(h-BN/g-C_3_N_4_) was constructed. The formed excellent interface contact between the materials found to greatly enhance the surface area and promoted the charge separation and transfer. As compared to the pristine g-C_3_N_4_, the photocatalytic activity of h-BN/g-C_3_N_4_ composite was found to be enhanced on the photocatalytic oxidation of tetracycline under visible light (300 W xenon lamp, 420 nm cut-off filter). The optimized composite 0.48 wt% in h-BN/g-C_3_N_4_ showed ~2.3 and 60.3-folds higher tetracycline degradation efficiency than the bare g-C_3_N_4_ and h-BN, respectively. The enhanced photocatalytic activity of h-BN/g-C_3_N_4_ composite was mainly attributed to the larger surface area and unique physicochemical properties of h-BN nanosheet, where the h-BN was acted as a promoter for photoexcited holes transfer. Besides, the photo-degradation process was dominated by the O_2_^•−^ and h^+^, while ^•^OH radicals was neglected [66]. The ternary g-C_3_N_4_/ZnTcPc/GQDs composite showed an excellent photocatalytic activity towards the complete degradation of sulfaquinoxaline sodium (less than 40 min) and carbamazepine (less than 180 min) under solar light irradiation using Q-Sun Xe-1 chamber. The incorporation of ZnTcPc with g-C_3_N_4_ broadened the visible-light photoabsorbance range and the uniform dispersion of GQDs onto g-C_3_N_4_ surface facilitated for the efficient electrons transfer. In addition, this system also showed excellent photocatalytic performance over a wide range of pH, and the presence of ^1^O_2_, O_2_^•−^ and h^+^ was identified as the main active species in the photodegradation process [67]. The g-C_3_N_4_@Ni-Ti layered double hydroxides (g-C_3_N_4_@Ni-Ti LDH NCs) nanocomposite with the high surface area was synthesized by the optimized hydrothermal process in the presence of NH_4_F. The as-synthesized composite materials were examined for sono-photocatalytic degradation of amoxicillin antibiotics under visible light irradiation, where the g-C_3_N_4_@Ni-Ti LDH NCs composite showed the better photocatalytic activity as compared to pure g-C_3_N_4_ and Ni-Ti LDH materials. The enhancement in the sono-photocatalytic performance of the nanocomposites was originated from their higher specific surface area, perfect interface contact and reduced electron-hole recombination. In addition, for the comparison, the bare sonolysis and bare photocatalysis processes also applied forthe degradation of amoxicillin [68]. Recently, the zeoliticimidazolate framework (ZIF) and semiconductor composites displayed excellent adsorption and catalytic performance, which provided a new way for the development of efficient hybrid photocatalysts for photocatalytic antibiotics removal. In particular, the zeoliticimidazolate framework-8 (ZIF-8) showed more desirable properties such as good stability and large surface area. For example, the photo-regenerable and bifunctional C_3_N_4_-ZIF-8 composite was developed for efficient adsorption and solar photocatalytic degradation of tetracycline (Figure 8). The bifunctional composite was established by anchoring the ZIF-8 micro crystals on C_3_N_4_ nanosheets, which led to the formation of highly stable micro-mesoporous architecture. The C_3_N_4_-ZIF-8 composite showed the high photo-regenerable adsorbent capacity of 420 mg g^−1^ and a higher rate of photocatalytic tetracycline removal of around 90% within 60 min under solar irradiation. The π-π and electrostatic interactions between the antibiotic molecules and the composite combined with appropriate pore configurations provided the high adsorption capacity. Further, the anchoring of C_3_N_4_ sheets on ZIF-8 microcrystal led to the efficient heterostructure formation and established better interfaces contact, which hindered photogenerated recombination [69].

Similarly, the highly efficient ZIF-8/g-C_3_N_4_ photocatalytic composite hindered the aggregation of g-C_3_N_4_ nanosheets and showed efficient adsorbent capturing capacity, which eventually increased the contacts between the active species and antibiotic molecules. The formation of ZIF-8/g-C_3_N_4_ composite significantly improved the visible light absorption and simultaneously boosted the charge transfer and separation of the photogenerated electron-hole pairs, thereby improved the photocatalytic efficiency. The synergistic effect of preparation method and special hybrid composite structure together significantly enhanced the photocatalytic degradation of tetracycline under visible light irradiation, which found to be 1.69 folds higher activity than that of pure g-C_3_N_4_ [70].

### 3.8. Indium Hybrids

The construction of heterojunction was found to be an effective way to enhance the photocatalytic performance of the semiconductor photocatalysts. For example, the In_2_S_3_/BiPO_4_ heterojunction composite photocatalyst was successfully fabricated via irregular loading of In_2_S_3_ onto the BiPO_4_ surface. The achieved well-dispersion of In_2_S_3_ greatly improved surface contact and active sites of the heterostructure composite. Besides, the In_2_S_3_ also greatly enhanced the visible light absorption in BiPO_4_. Therefore, the as-developed In_2_S_3_/BiPO_4_ heterojunction composite showed higher visible light photocatalytic activity for the photodegradation of tetracycline (71.0% in 100 min) as compared to those of pure In_2_S_3_ and BiPO_4_. Moreover, the electron spin resonance (ESR) technique and active species trapping experiments indicate that the O_2_^•−^ and h^+^ were the main active species in the photodegradation process [71]. Similarly, the novel 3D microsphere-like In_2_S_3_/InVO_4_ heterojunction composite was fabricated via a simple in-situ anion exchange method by the treatment of Na_2_S with pre-synthesized InVO_4_ microspheres. The as-developed In_2_S_3_/InVO_4_ microspheres showed ~2.26 and ~11.71 folds higher tetracycline degradation as compared to In_2_S_3_ and InVO_4_ under visible-light irradiation (300 W xenon lamp, 420 nm cutoff filter). The obtained superior photocatalytic performance was attributed to the enhanced photo-absorption in the visible region by photosensitization of InVO_4_ with In_2_S_3_. Then, the formed close interface contacts between the In_2_S_3_ and InVO_4_ established greater charge separation and reduced recombination of photogenerated electron-hole pairs [72]. The rapid recombination of electrons and holes in a single photocatalyst largely limited its performance. For instance, the development of an In_2_S_3_/NaTaO_3_ heterojunction composite potentially improved the photocatalytic efficiency as compared to the single component NaTaO_3_ and In_2_S_3_ photocatalyst towards the degradation of tetracycline hydrochloride under simulated solar irradiation [73]. Similarly, the controlled synthesis of Z-scheme InVO_4_/CdS heterojunction composite displayed an enhanced photocatalytic activity towards the degradation of ciprofloxacin as compared to pure InVO_4_ and CdS under visible light irradiation (λ > 420 nm). The improved photocatalytic activity was attributed to the Z-scheme heterojunction system with enhanced electron-hole pair separation, tran sfer and stability [74].

### 3.9. Iron Hybrids

**Figure 9:**
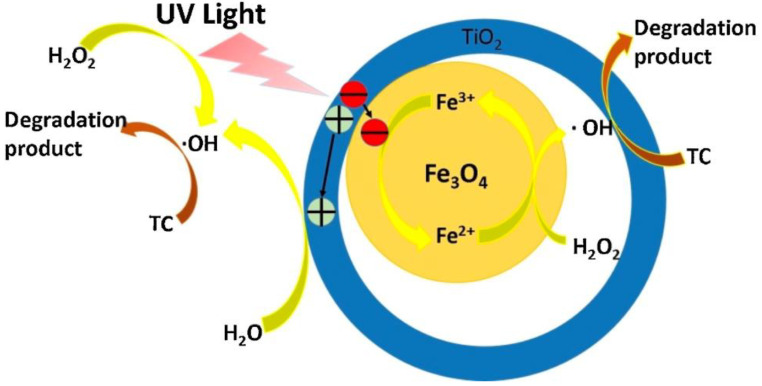
The photo-Fenton-like degradation mechanism for tetracycline [76].

Recently, the magnetic ultrathin γ-Fe_2_O_3_ nanosheets/mesoporous black TiO_2_ hollow sphere (γ-Fe_2_O_3_/b-TiO_2_) heterojunctions were fabricated via metal-ion intervened hydrothermal technique and high-temperature hydrogenation, which demonstrated an enhanced solar photocatalytic degradation of tetracycline (99.3% after 50 min) under AM 1.5 irradiation. The surface hydrogenation process converted the α-Fe_2_O_3_ nanosheets into γ-Fe_2_O_3_ ultrathin nanosheets (∼6 nm) with the formation of surface defects (oxygen vacancy). The formation of oxygen vacancy and Ti^3+^ in black TiO_2_ frameworks was found to be beneficial for the efficient solar-light-harvesting, and the ultrathin nanosheet and hollow structure favored to the diffusion and transportation of photogenerated charge carriers in the γ-Fe_2_O_3_/b-TiO_2_ heterojunction composite. Besides, the apparent degradation rate constant (k) of γ-Fe_2_O_3_/b-TiO_2_ was ∼3 times higher than that of α-Fe_2_O_3_/b-TiO_2_ under AM 1.5 irradiation [75]. Similarly, the yolk-shell structured Fe_3_O_4_@void@TiO_2_ NPs composite exhibited well-defined hollow structure and high specific surface area, which remarkably augmented the photocatalytic performance towards the degradation of tetracycline. The composite showed extremely higher activity towards the degradation of tetracycline (40 ppm) in a wide range of pH as demonstrated almost 100% removal efficiency at pH = 3 within 6 min. The degradation curve well fitted by pseudo-first-order model, the kinetic constant of the Fe_3_O_4_@void@TiO_2_ reached 0.51 min^−1^, which was much higher than those of Fe_3_O_4_@TiO_2_ (0.24 min^−1^), hollow TiO_2_ (0.17 min^−1^), Fe_3_O_4_@SiO_2_@TiO_2_ (0.14 min^−1^) and Fe_3_O_4_ (0.11 min^−1^). The superior activity was attributed to the efficient enrichment and confinement of reactants (tetracycline and hydroxyl radicals) in the nanocavity of the yolk-shell structure and the efficient reduction of Fe^3+^ to Fe^2+^ was by the photo-generated electrons from the TiO_2_ shell (Figure 9). Then, the superparamagnetic and good stability of Fe_3_O_4_@void@TiO_2_ showed a convenient separation of catalyst along with good recyclability [76].

The magnetically separable graphene oxide supported titanium dioxide integrated ferro-ferric oxide (Fe_3_O_4_/rGO/TiO_2_) hybrid photocatalyst enhanced the photocatalytic activity towards the degradation of tetracycline hydrochloride (~92.6%) under visible light irradiation. The enriched catalytic activity was achieved by the synergistic effect of photo-Fenton reaction and the higher charge transportation and conducting ability of graphene. Furthermore, the catalyst system is found to be with high reusability and excellent photostability [77]. The ZnO/α-Fe_2_O_3_ nanoflowers were potentially prepared by reflux and hydrothermal method, where their morphological formation was due to the assembly of many nanosheets like structures. The photocatalytic activity of the composite was investigated on the photo-degradation of cefiximetrihydrate under UV-vis irradiation and it showed a maximum degradation efficiency of 99.1% in 130 min [78]. Further, the functional photocatalyst based on ZnO/C/Fe_3_O_4_ heterojunction showed a strong three-dimensional oriented selectivity on recognition of antibiotic danofloxacinmesylate and as well as higher photocatalytic degradation efficiency. The observed photocatalytic reaction was mainly contributed to the h^+^ and ^•^OH species. In addition, this functional system also showed hollow capsule structure, good light response-ability, superior magnetic separation and excellent reproducibility [79]. The new FeVO_4_/Fe_2_TiO_5_ heterojunction composite exhibited higher visible light photocatalytic activity towards the degradation of norfloxacin (~92.0% of degradation and 49.62% of mineralization) and excellent photo-stability. The achieved higher photocatalytic activity was attributed by the synergistic effect of photogenerated electron-hole with holes and hydroxyl radicals. It was observed that the degradation process was initiated by breaking the piperazine ring within norfloxacin by the generated reactive species [80].

### 3.10. Lanthanum Hybrids

**Figure 10:**
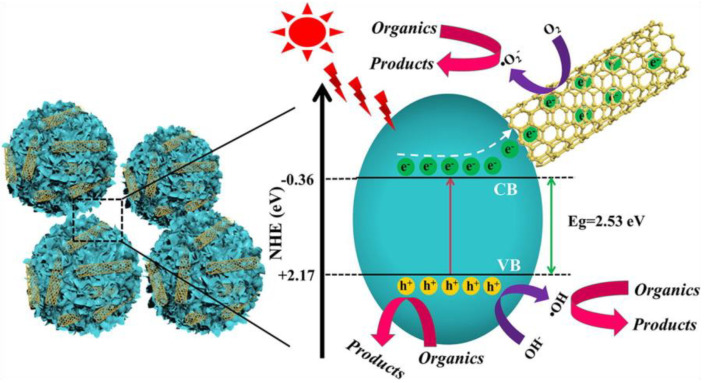
Schematic diagram of the photocatalyti cmechanism of CNT/LaVO_4_ composite [85].

The n-n heterojunction based on La(OH)_3_/BiOCl composite system with oxygen vacancy was developed and it degraded around 85% of tetracycline hydrochloride in 60 min under visible light irradiation using 5 W LED light (λ ≥ 420 nm), which was 0.5 and 2 folds higher than that of bare La(OH)_3_ and BiOCl. This heterostructure induced a reassembled internal electric field and oxygen vacancies (OVs) in the system, which intrinsically broadened the photo-responsive range and increased the separation of photogenerated carriers [81]. Similarly, the rationally designed La(OH)_3_@BaTiO_3_ Z-scheme core-shell heterostructure possessed high negative conduction band, where it led to the complete degradation of A-ring of tetracycline. Notably, it was more difficult to degrade the A-ring of tetracycline completely as compared to the complete degradation of B-D rings of tetracycline. Moreover, the active species trapping experiments showed that the O_2_^•−^ species were more responsible for the degradation of A-ring in tetracycline. In addition, Z-scheme was also found to improve the lifetime of the photoinduced charge carriers, thereby higher photocatalytic properties [82]. The rationally designed Z-scheme LaNiO_3_/g-C_3_N_4_ hybrids showed strong interface contact and remarkable photocatalytic performance towards the tetracycline degradation under visible light irradiation (λ > 420 nm). This hybrid demonstrated ~3.9 and 3.8 folds higher photocatalytic performance than that of pristine LaNiO_3_ and g-C_3_N_4_. Furthermore, this Z-scheme construction was not only facilitated efficient charge transfer mechanism between LaNiO_3_ and g-C_3_N_4_, but it also endowed strong redox ability in the LaNiO_3_/g-C_3_N_4_ hybrids. Furthermore, active species trapping experiments revealed that the synergistic effect of superoxide radicals and holes was responsible for the photodegradation of tetracycline [83]. The binary LaCoO_3_/Ag_2_CrO_4_ hybrid photocatalyst showed a significantly higher rate of tetracycline degradation (0.0121 min^−1^) under visible light, where it was found to be around 3.6 and 8.4 folds better as compared to LaCoO_3_ (0.0033 min^−1^) and Ag_2_CrO_4_ (0.0014 min^−1^), respectively. The LaCoO_3_ with excellent charge conductivity and higher charge mobility were attributed to the observed enhancements. Moreover, the LaCoO_3_ increased the photogenerated charge carriers separation by rapid capturing of photoexcited electrons from Ag_2_CrO_4_ and as a result, it suppressed the photo-corrosion in Ag_2_CrO_4_ as well [84]. The rational construction of stable CNT/LaVO_4_ nanostructures presented an efficient photocatalytic performance, where it degraded ~81% of tetracycline in 180 min, which was 2 times higher than that of pure LaVO_4_ (40.0%). The incorporation of 0.1 wt% CNT with LaVO_4_ has potentially increased the photogenerated charge carriersseparation and transfer of and as well as degradation efficiency (Figure 10). Besides, the antibacterial results showed that the degraded products were lower in toxicity [85]. Similarly, the carbon nanotubes incorporated lanthanum vanadate (CNT/LaVO_4_) with different mass ratios were explored on the sulfamethazine degradation. The experimental result showed that the 0.3% CNTs/LaVO_4_ (70%) composite found higher photocatalytic performance than 0.1% CNTs/LaVO_4_ (66%) and LaVO_4_ (33%) under the optimum reaction condition. The higher activity of 0.3% CNTs/LaVO_4_ composite was attributed to the enhancement of adsorption properties of LaVO_4_ by addition of CNTs, and also photogenerated electrons of CNTs was migrated to the conduction band of LaVO_4_, which resulting in the formation of highly reactive superoxide radicals and hydroxyl radicals. In addition, CNTs employed as an electron acceptor or donor and facilitated to reduce the recombination rates [86].

### 3.11. Lead Hybrids

The flower-like porous CNT/PbBiO_2_Br hybrid composite was developed through the uniform distribution of CNT on PbBiO_2_Br nanosheets surface and studied for ciprofloxacin removal under UV, visible and above 580 nm light. The introduction of CNT improved the photocatalytic activity of CNT/PbBiO_2_Br composites, which mainly attributed to the enhanced light capture capability and superior charge transferability. Moreover, CNT act as photoinduced electron separation center as well as pollutant degrading activity center. Therefore, the CNT/PbBiO_2_Br composite showed a superior photocatalytic performance (88% in 150 min) as compared to the pristine PbBiO_2_Br (27% in 150 min) [87]. The MoS_2_/PbBiO_2_I hybrid composite developed by attaching the microsphere like the structure of PbBiO_2_I nanosheets with MoS_2_, where this system showed enhanced degradation efficiency towards ciprofloxacin under visible light irradiation (300 W xenon lamp with cut-off filter λ > 400 nm). It was observed that the suitable band structure facilitated higher photocatalytic activity in MoS_2_/PbBiO_2_I (84.0% of degradation) as compared to pure PbBiO_2_I (46.0% of degradation). In addition, the strong interface interaction between MoS_2_ and PbBiO_2_I offered larger specific surface area, enhanced light absorption and stronger photocurrent intensity in MoS_2_/PbBiO_2_I composite, thereby showed effective electron-hole pair separation and more active sites generation [88]. The zeolite bed played an important role in the photocatalytic activity, therefore clinoptilolite nanoparticles supported CdS-PbS hybrids were developed and explored for the degradation of a mixture of antibiotics tetracycline and cephalexin under UV irradiation (with 2 UV tubes 35 W). The clinoptilolite (zeolite) incorporation prevented the aggregation of CdS-PbS particles and also improved electron-hole separation towards improved photocatalytic activity than that of the unsupported CdS-PbS photocatalyst. Moreover, the effect of inorganic salts such a NaCl and Na_2_CO_3_ and organic compound isopropanol were studied on the degradation of the antibiotic [89]. A wide-spectrum-responsive plasmonic Ag/Ag_2_O/PbBiO_2_Br p-n heterojunction photocatalyst was developed and it revealed 84.4% degradation of tetracycline under visible light (300 W Xe lamp, 420 nm filter) and 50.9% degradation under NIR light (300 W Xe lamp, 800 nm filter) irradiation. The observed boosted photocatalytic activity was ascribed to the synergistic effect between plasmonic Ag and Ag_2_O/PbBiO_2_Br p-n heterojunction, which significantly broadened the light absorbance and also accelerated the charge separation. In addition, O_2_^•−^, h^+^ and ^•^OH radicals were identified as major reactive species involved in the degradation of tetracycline under visible light irradiation, while O_2_^•−^ and h^+^ were identified as major reactive species under NIR light [90].

### 3.12. Manganese Hybrids

The triethoxysilane and guanidine nitrate modified MnO_2_-NiO nanocomposites were developed with enhanced nanocarriers and enhanced surface properties. This developed MnO_2_-NiO nanocomposite was found to degrade 89.55% of tetracycline within 40 min under UV light (125 W, λ = 365 nm), which was attributed to the synergistic effect between MnO_2_ and NiO towards forming the Ni-Mn-O structure [91]. Similarly, the 2D/2D MnIn_2_S_4_/g-C_3_N_4_ Z-scheme nanocomposites presented almost complete degradation of tetracycline hydrochloride within 90 min under visible light irradiation (300 W xenon lamp, 400 nm cut-off filter). The Z-scheme heterogeneous architecture enabled a fast charge carrier separation originated due to their strong interfacial contacts and well-matched band structures. In addition, the cyclic photocatalytic test revealed excellent stability of the MnIn_2_S_4_/g-C_3_N_4_ nanocomposites [92]. The graphitic carbon sand composite (GSC) and bentonite (BT) supported MnFe_2_O_4_ superparamagnetic MnFe_2_O_4_/GSC and MnFe_2_O_4_/BT composites were successfully developed and employed for the effective removal and degradation of ampicillin and oxytetracycline by both adsorption and photocatalytic processes. Accordingly, the MnFe_2_O_4_/GSC and MnFe_2_O_4_/BT removed around 96 and 83% of ampicillin within 60 min and 99.0 and 90% oxytetracycline in 120 min under solar light irradiation, respectively. Furthermore, the magnetic MnFe_2_O_4_/GSC and MnFe_2_O_4_/BT displayed significant photocatalytic efficiency and recoverability for up to ten cycles without any loss of catalysts [93]. Similarly, the magnetic g-C_3_N_4_/MnFe_2_O_4_/graphene (C_3_N_4_@MnFe_2_O_4_-G) composites with improved photo Fenton-like degradation were studied for the degradation antibiotics metronidazole, amoxicillin, tetracycline and ciprofloxacin using persulfate (S_2_O_8_^2−^) as an oxidant under visible light illumination (300 W Xe lamp, 400 nm cutoff filter). The formation of the heterojunction between g-C_3_N_4_ and MnFe_2_O_4_ increased the photo-absorption capacity as well as improved charge carriers migration and lifetime. Accordingly, the developed nanocomposites showed superior catalytic efficiency of ~94.5% metronidazole degradation. Besides, the self-redox properties of iron and manganese atoms in MnFe_2_O_4_ that induced by S_2_O_8_^2−^ were particularly beneficial for the generation of SO_4_^•−^ radicals [94]. The superior Mn_2_O_3_/Mn_3_O_4_/MnO_2_ photocatalyst with dual heterostructure was designed from Oxone induced Mn_2_O_3_. The Mn_2_O_3_/Mn_3_O_4_/MnO_2_ heterojunction demonstrated a higher photocatalytic performance on the degradation of ciprofloxacin (95.6% of degradation and 63.9% of mineralization) under visible light irradiation (300 W Xe lamp with a light intensity of 900 mW/cm^2^). The outstanding catalytic performance was attributed to their improved surface area, decreased isoelectric point, enhanced light absorption and efficient charge separation. Further, the developed Mn_2_O_3_/Mn_3_O_4_/MnO_2_ heterojunction showed the selective degradation of ciprofloxacin and offered high practical reusability [95].

### 3.13. Molybdenum Hybrids

**Figure 11:**
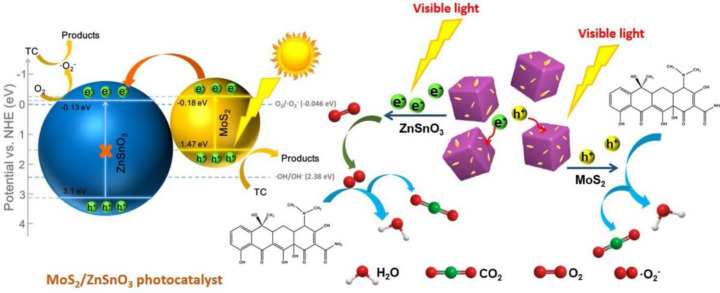
Photocatalytic mechanism of MoS_2_/ZnSnO_3_ composite for degradation of tetracycline under visible light irradiation [97].

The Z-scheme MoS_2_/Bi_2_O_3_ heterojunction was developed by coupling the MoS_2_ nanosheets onto the surface of Bi_2_O_3_ rods, where the developed MoS_2_/Bi_2_O_3_ composite showed around 4.26 and 1.94 times higher photocatalytic performance than that of the pure MoS_2_ and Bi_2_O_3_ towards the degradation of tetracycline under visible light irradiation. The achieved enhanced photocatalytic activity was attributed to the combination of Bi_2_O_3_ and MoS_2_ and their extended photo-absorption and the tight interface connection with a good energy band match in the formed heterojunction. In addition, the efficient interfacial interaction between MoS_2_ and Bi_2_O_3_ accelerated photoinduced charge carriers separation and also enlarged the specific surface area [96]. A composite system based on MoS_2_/ZnSnO_3_ was developed by loading the MoS_2_ nanosheets over the surface of porous ZnSnO_3_ microcubes. This synthesized composite demonstrated outstanding photocatalytic performance on the degradation of tetracycline over 80% and mineralization over 42% within 60 min under visible light (300 W xenon lamp with UV cut-off filters). The observed remarkable photocatalytic tetracycline degradation was attributed to their broadened photo-harvesting and effective photoinduced charge carrier separation efficiencies. The in-depth investigation on the photocatalytic mechanism of MoS_2_/ZnSnO_3_ composite under visible light is depicted in Figure 11 [97].

**Figure 12:**
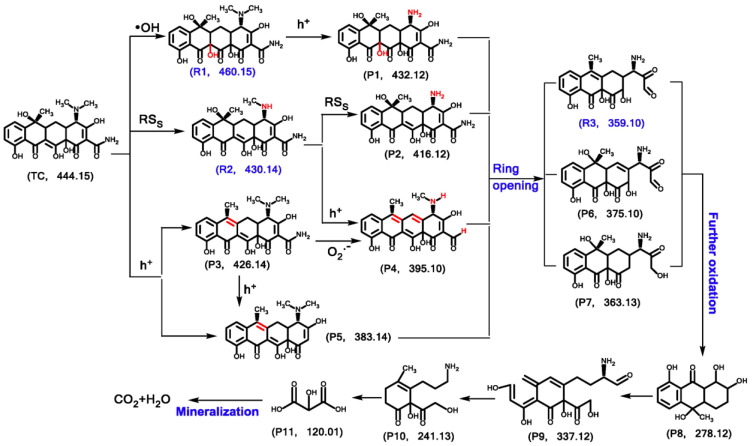
The proposed degradation pathway and intermediates of tetracycline [100].

The 1D Ag_2_Mo_2_O_7_ microrod decorated 2D MoS_2_ nanosheets based 1D/2D Z-heterostructure composite was developed and demonstrated for the enhanced photocatalytic degradation of levofloxacin. The as-developed heterostructure composite showed an improved photocatalytic performance of around 97% levofloxacin degradation in 90 min under visible-light irradiation (150 W Xe lamp, AM 1.5 G filter). The higher activity was ascribed to the well-aligned and favorable band positions, which allowed the efficient charge transfer between the photogenerated charge carriers, and the direct Z-scheme led to a large number of photogenerated electrons at the MoS_2_ surface. Furthermore, the radical-trapping experiments confirmed that the super oxide and hydroxyl radicals were the key species participated in the photodegradation. Moreover, the reusability tests displayed the higher stability of the developed composite [98]. The Z-scheme system based on MoO_3_/Ag/C_3_N_4_ composite was developed and it showed the excellent visible-light photocatalytic activity for degradation of fluoroquinolone antibiotic ofloxacin and found to show 3 folds higher degradation rate as compared to MoO_3_/C_3_N_4_ and Ag/C_3_N_4_. The observed enhanced photocatalytic efficiency was attributed the Z-scheme mechanism, where the Ag nanoparticles in the MoO_3_/Ag/C_3_N_4_ composite were acted as a mediator to accelerate the charge transfer through MoO_3_ and C_3_N_4_, and boosted the generation of hydroxyl radicals in the degradation process [99]. Similarly, the carbon dots modified-MoO_3_/g-C_3_N_4_ Z-scheme heterostructure photocatalyst was developed and it showed around 88.4% degradation of tetracycline in 90 min under visible light (350 W xenon lamp, 420 nm cutoff filter), which was 3.5 folds higher than that of MoO_3_/g-C_3_N_4_. The synergetic effect of CDs (act as electron reservoir and optical converter) and Z-scheme heterojunction together enhanced the electron-hole pair separation and electron transfer, thereby improved the photocatalytic activity of CDs/g-C_3_N_4_/MoO_3_ system. The possible intermediates of tetracycline were identified, and their degradation pathway was also proposed as shown in Figure 12 [100].

Interestingly, a highly efficient visible-driven molybdenum disulfide/zeoliticimidazolate framework (MoS_2_/ZIF-8) composite was developed and studied for the degradation of ciprofloxacin and tetracycline. This composite was found to have 1.21- and 1.07-folds higher efficiencies as compared to the pure MoS_2_ under visible light irradiation (300 W Xe lamp, 420 nm cut-off filter). The mixed phase of MoS_2_ augmented the electron conductivity and expanded density of active sites in the system thereby it increased the electron transfer and mass transfer efficiency. Furthermore, the ultrathin long tubular structure of MoS_2_ provided the fast photoexcited electrons transfer and reduced the charge recombination and thereby improved the overall photocatalytic performances [101].

### 3.14. Nickel Hybrids

**Figure 13:**
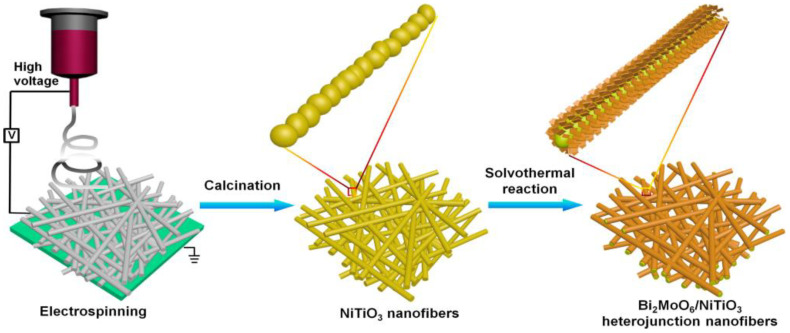
Electrospinning preparation of Bi_2_MoO_6_/NiTiO_3_ nanofibers [105].

The system consisted of magnetic NiFe_2_O_4_/graphitic carbon nitride (GCN/NiFe_2_O_4_) was demonstrated for the mineralization of oxytetracycline under solar light. Interestingly, the developed GCN/NiFe_2_O_4_ composite completely mineralized the oxytetracycline in 8 h through simultaneous adsorption and degradation process in optimum pH. The achieved excellent photocatalytic activity was attributed to high surface area and efficient photogenerated electron-hole separation inGCN/NiFe_2_O_4_ heterostructure. In addition, the ferromagnetic property of GCN/NiFe_2_O_4_ composite offered an easy recovery of the catalyst and significant recyclability efficiency [102]. Similarly, the magnetically separable NiFe_2_O_4_/Bi_2_O_3_ heterostructure was found to show an enhanced visible-light driven photocatalytic activity for the degradation of tetracycline and reached ~91% in 90 min using a 150 W xenon lamp with UV cut-off filter. The well-matched band structure led to an efficient charge separation and transfer across the interface of the heterostructure, thereby improved the photocatalytic performance of NiFe_2_O_4_/Bi_2_O_3_ heterostructures. Further, the developed photocatalyst was recovered and recycled under a magnetic field along with good stability [103]. Similarly, the magnetically recoverable system composed of carbon dots (CDs) and NiCo_2_O_4_ photocatalyst was developed and showed enhanced photocatalytic activity towards the degradation of tetracycline under visible-light irradiation, where the 3 wt% CDs/NiCo_2_O_4_ (0.0213 min^−1^) found to show 6 folds higher degradation efficiency as compared to the pristine NiCo_2_O_4_ (0.0036 min^−1^). This observed improved photocatalytic activity of CDs/NiCo_2_O_4_ composite was assigned to the synergetic effect of CDs and NiCo_2_O_4_, which improved both the light absorption capacity as well as photo-induced charge carrier separation efficiency [104]. Likewise, a rationally designed system composed of highly dispersed Bi_2_MoO_6_ nanosheets that anchored on the electrospun NiTiO_3_ nanofibers was developed to construct the Bi_2_MoO_6_/NiTiO_3_ heterojunction (Figure 13) towards the efficient degradation of tetracycline hydrochloride under visible light (300 W xenon lamp, λ > 400 nm). The observed favorable interfacial contact and well-matched band structure in Bi_2_MoO_6_/NiTiO_3_ were found to suppress their combination of photo-generated electron-hole pairs. As a result, the stable Bi_2_MoO_6_/NiTiO_3_ heterojunction composite showed 26, 5.4 and 3.7 folds higher photodegradation rate constant (k) as compared to the pristine NiTiO_3_, Bi_2_MoO_6_ and mechanically mixed Bi_2_MoO_6_/NiTiO_3_ composite. Moreover, the rationally designed heterojunction composite revealed effective mineralization of tetracycline and effective recyclability [105].

The magnetite polypyrrole core-shell (Fe_3_O_4_@PPY) immobilized NiS nano-photocatalyst was developed by the in-situ chemical oxidative polymerization process. The as-developed Fe_3_O_4_@PPY-NiS nano-photocatalyst explored for the degradation of cephalexin and found to show 100 and 85% degradation efficiency under UV and solar light irradiation, respectively. The NiS immobilization on PPY-Fe_3_O_4_ led to suppress the recombination of the photogenerated electron-hole pairs and extended the photoabsorbance. Accordingly, the magnetite polypyrrole immobilized NiS was found to show higher photocatalytic activity and stability as compared to the bulk NiS [106].

### 3.15. Silver Hybrids

It has been well demonstrated that the noble metals NPs exhibit the surface plasmon resonance (SPR) phenomenon, which offers strong and broad absorption in the visible region. Accordingly, the plasmonic Ag/Ag_2_MoO_4_ nanocomposite showed significantly improved photocatalytic activity towards degradation of ciprofloxacin under visible light due to the SPR effect of the Ag nanoparticles. As a result, the Ag/Ag_2_MoO_4_ composite degraded around 99.5% of ciprofloxacin in 60 min and showed excellent stability as compared to the bare Ag_2_MoO_4_. In addition, the active species h^+^ and ^•^OH were identified as the main reactive species in the photodegradation of ciprofloxacin [107]. The Ag_3_PO_4_/WO_3_ composites with different molar ratios were developed and studied for sulfamethoxazole degradation under simulated solar light. The Ag_3_PO_4_/WO_3_ (75:25) composite showed the highest activity of 90% sulfamethoxazole conversion achieved in just 2 min with an initial concentration of 525 μg/L of sulfamethoxazole and 200 mg/L photocatalyst. The result showed that the degradation efficiency was found to increase with increasing photocatalyst concentration from 25 to 200 mg/L and decrease the sulfamethoxazole concentration from 2100 to 260 μg/L. Besides, the degradation rate of antibiotic was faster in ultrapure water than antibiotic in bottled water and domestic wastewater. However, the domestic wastewater containing certain matrix constituents such as chloride, bicarbonate and humic acid showed some positive effect on sulfamethoxazole degradation [108]. A highly efficient functionalized AgBr/Ag_2_CO_3_ visible-light-driven photocatalyst was synthesized by facile ion-exchange technique. This developed hybrid photocatalyst exhibited excellent photocatalytic performance and photostability towards the degradation of tetracycline under visible light (λ > 420 nm) as compared to pure Ag_2_CO_3_ and AgBr [109]. Further, the metallic silver incorporated AgBr/Ag@Ag_2_O/Ag_2_CO_3_ multi-heterojunction composite was fabricated by simple precipitation assisted post calcination techniques and showed excellent photocatalytic activity for the degradation of ciprofloxacin under visible light (300 W Xe lamp, cut-off filter 420 nm). The photodegradation efficiency reached 44.8, 49.6, 64.1, 71.6 and 89.3%, when the corresponding initial concentration of ciprofloxacin was 50, 40, 30, 20 and 10 mg/L, respectively. The formation of multi-heterojunction was leading to higher charge carrier separation and excellent catalytic performance. In addition, the calcination temperatures and timings were also found to influence on the phase formation of Ag_2_O in the system [110]. The advanced system composed of Ag/AgCl/Ag_2_O was developed by growing the Ag/AgCl on the surface of Ag_2_O nanoparticles at room temperature. Interestingly, this system was found to overcome the drawbacks of Ag_2_O and provide strong redox ability and long-term stability. The established Ag/AgCl nanoshells were effectively protected the core Ag_2_O particles from photo corrosion and improved the charge carrier separation and transfer efficiency. The optimum composite was found to potentially degrade the high resistant antibiotic ciprofloxacin under visible light irradiation (λ > 420 nm). The obtained results showed that the photocatalytic efficiency of Ag/AgCl/Ag_2_O heterostructure was about 2.9 and 3.73 times higher than that of Ag_2_O and Ag/AgCl catalyst [111].

**Figure 14:**
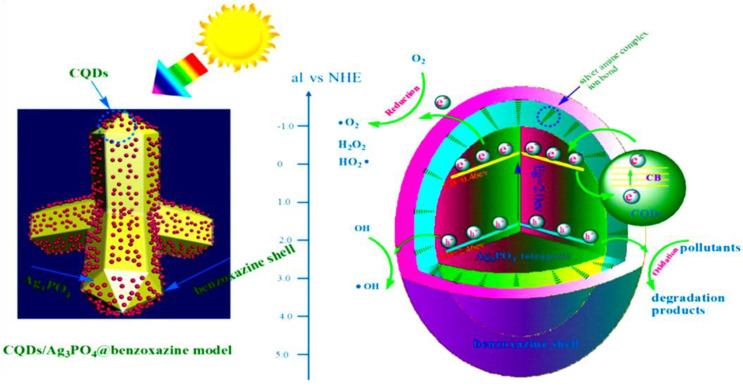
Schematic illustration of the energy band structure and charge transfer mechanism of 3D CQDs/Ag_3_PO_4_@benzoxazine composites [113].

Similarly, the designing of reduced graphene oxide (RGO) enwrapped TiO_2_ nanobelts supported Ag_2_O nanocomposite based solid-state Z-scheme photocatalytic system was found to suppress the photo-corrosion and promote the charge separation in the Ag_2_O system. Meanwhile, the RGO incorporation between Ag_2_O and TiO_2_ potentially improved the transfer of photogenerated electrons from Ag_2_O to TiO_2_ and prolonged their lifetime through Z-scheme mechanism. Further, the RGO-Ag_2_O/TiO_2_ composite was potentially investigated on the degradation of tetracycline under UV light, visible light, near-infrared (NIR) light and simulated solar light irradiation [112]. The CQDs and benzoxazine modified Ag_3_PO_4_ was exploited to develop a 3D core-shell CQDs/Ag_3_PO_4_@benzoxazine tetrapod composite system, which found to improve the photocatalytic activity and photostability of Ag_3_PO_4_. Especially, CQDs in the system promoted the generation of charge carriers and improved the charge transfer from Ag_3_PO_4_ to CQDs, where the silver-amine complex acted as a bridge for the photoelectrons to flow from the core to shell (Figure 14). Therefore, the as-developed CQDs (0.38%)/Ag_3_PO_4_@benzoxazine tetrapod composite displayed excellent photocatalytic activity for degradation of sulfamethoxazole, where it showed around 95% degradation within 15 min under visible light with 800 W xenon arc lamp irradiation. Moreover, the 3D core-shell structure selectively suppressed the photo-corrosion in Ag_3_PO_4_ and thereby improved the stability and reusability of the catalyst was achieved [113].

More interestingly, the metal carbide Ti_3_C_2_ potentially improved the photocatalytic efficiency and photo-stability of Ag_3_PO_4_ towards the degradation of tetracycline. The Ti_3_C_2_ established a strong interfacial contact, which facilitated the formation of potential Schottky junction between Ag_3_PO_4_ and Ti_3_C_2_, thereby it improved the separation of charge carriers, photocatalytic efficiency and stability of the catalyst [114].

### 3.16. Strontium Hybrids

**Figure 15:**
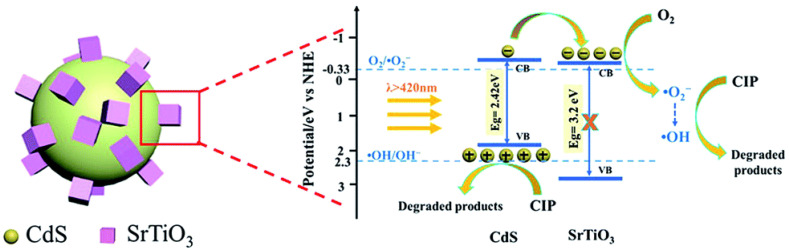
Photocatalytic degradation mechanism of ciprofloxacin over CdS/SrTiO_3_ heterojunction under visible light [118].

The SrTiO_3_/Bi_2_O_3_ heterostructure photocatalysts showed an improved visible photocatalytic activity of 85% degradation of tetracycline in 140 min, which was higher than that of the pristine SrTiO_3_ and Bi_2_O_3_. The enhanced photocatalytic activity was ascribed to the efficient interface contact and heterojunction formation between SrTiO_3_ and Bi_2_O_3_, which greatly improved the separation and transfer of photoinduced charge carriers at the two-phase interface of the heterojunction composite with higher surface area [115]. This novel BiVO_4_/SrTiO_3_ heterojunction composite showed an excellent photocatalytic performance towards the degradation of sulfamethoxazole under the irradiation of xenon lamp, where it showed around 91% degradation and around 48% mineralization of sulfamethoxazole within 60 min. The achieved superior photocatalytic activities were attributed to the heterojunction construction and its enhanced surface area of the composite. Moreover, it was predicted that the mechanism of degradation involved the cleavage of C–O, C–S, C–C, C–N, isoxazole and benzene ring in sulfamethoxazole molecules [116]. The visible-light-driven Cu_2_O/SrTiO_3_ p-n heterojunction photocatalyst was developed via the incorporation of Cu_2_O nanoparticles (~5 nm) on SrTiO_3_ nanocubes (~50 nm) by facile deposition-precipitation technique. The Cu_2_O/SrTiO_3_ heterojunction photocatalyst was employed for the photodegradation of tetracycline and it showed the highest catalytic efficiency of ~78% degradation in 100 min under visible light (150 W Xe lamp, cut off light λ < 420 nm). The observed higher photocatalytic activity was due to the fast migration of photogenerated electrons from Cu_2_O to SrTiO_3_; thereby there was an improved electron-hole charge separation in the composite. Further, this system also demonstrated the possibility of replacing the low-cost Cu_2_O nanoparticles instead of noble metals towards improving their photocatalytic ability towards the degradation of antibiotic molecules [117]. The CdS/SrTiO_3_ heterojunction based on CdS/SrTiO_3_ showed an improved photocatalytic activity for ciprofloxacin degradation (93.7% in 120 min) under visible light irradiation. The observed higher photocatalytic was attributed to the formation of the heterostructure, which augmented the separation efficiency of photogenerated electrons and holes (Figure 15). In addition, the CdS/SrTiO_3_ heterojunction demonstrated an excellent photocatalytic activity towards the degradation of multiple antibiotics such as enrofloxacin hydrochloride (91.1%), oxytetracycline (90.3%), danofloxacinmesylate (91.5%) and levofloxacin (88.6%) under visible light irradiation (250 W Xe lamp with a cut-offfilter at 400 nm) [118].

The rational designing of highly active SrTiO_3_/g-C_3_N_4_ heterojunctions bridged with Ag/Fe_3_O_4_ was performed to develop SrTiO_3_/(Ag/Fe_3_O_4_)/g-C_3_N_4_ ternary composite and employed for photodegradation of levofloxacin under ultraviolet, visible, near infra-red and natural solar light. The composite showed higher activity of 99.3% degradation of levofloxacin in 90 min under visible light. The binary heterojunction construction and topological properties of the system led to the improved charge carrier separation and reduced charge recombination, thereby the greater redox ability in the composite system. In addition, the synergistic effect of SrTiO_3_, g-C_3_N_4_ and plasmon resonance of Ag/Fe_3_O_4_ collectively improved the photoabsorption properties of the system. Moreover, the O_2_^•−^ and ^•^OH were observed as the main active radicals in visible light, whereas O_2_^•−^ was mainly generated under UV light [119].

### 3.17. Tin Hybrids

Zero-dimensional LaCoO_3_ nanoparticles decorated two-dimensional SnS_2_ nanosheets were employed for the photocatalytic degradation of tetracycline under visible light irradiation. The optimized 10 wt% LaCoO_3_ modified-SnS_2_ hybrid composite (0.0049 min^−1^) showed up to 7 folds higher rate of photocatalytic activity than the unmodified SnS_2_ (0.0007 min^−1^). The remarkable enhancement was attributed to LaCoO_3_ nanoparticles, which effectively captured the photogenerated electrons from SnS_2_ and boosted up the charge carriers separation and transfer [120]. Similarly, the mesoporous-Sn_3_O_4_/g-C_3_N_4_ Z-scheme heterostructure composite revealed a superior visible-light photocatalytic activity for the removal of tetracycline, where it showed degradation and mineralization of 72.2 and 61.2% of tetracycline, respectively in 120 min. The formation of Z-scheme heterostructure between Sn_3_O_4_ and g-C_3_N_4_ effectively improved the charge separation and suppressed the charge carrier recombination; thereby it improved the overall photocatalytic performance of the system. Besides, the mesoporous structure and enhanced specific surface area of Sn_3_O_4_/g-C_3_N_4_ composite possessed an abundant number of active sites for the effective adsorption and degradation of tetracycline molecules [121]. On the other hand, the construction of the p-n junction is found to tremendously promote the separation of electron-hole pairs and remarkably improve the overall photocatalytic performances. For example, the SnO_2_/BiOI n-p junction demonstrated a superior photocatalytic activity on the degradation of oxy-tetracycline hydrochloride (~94% in 90 min) under visible-light irradiation. The remarkable photocatalytic performance was found to be the construction of p-n junction of SnO_2_/BiOI and their band alignments, which effectively accelerated the electron-hole separation. Moreover, the reactive species h^+^ and O_2_^•−^ were found to be the key redox species responsible for the effective degradation by this SnO_2_/BiOI n-p junction hybrid composites [122].

### 3.18. Titanium Hybrids

**Figure 16:**
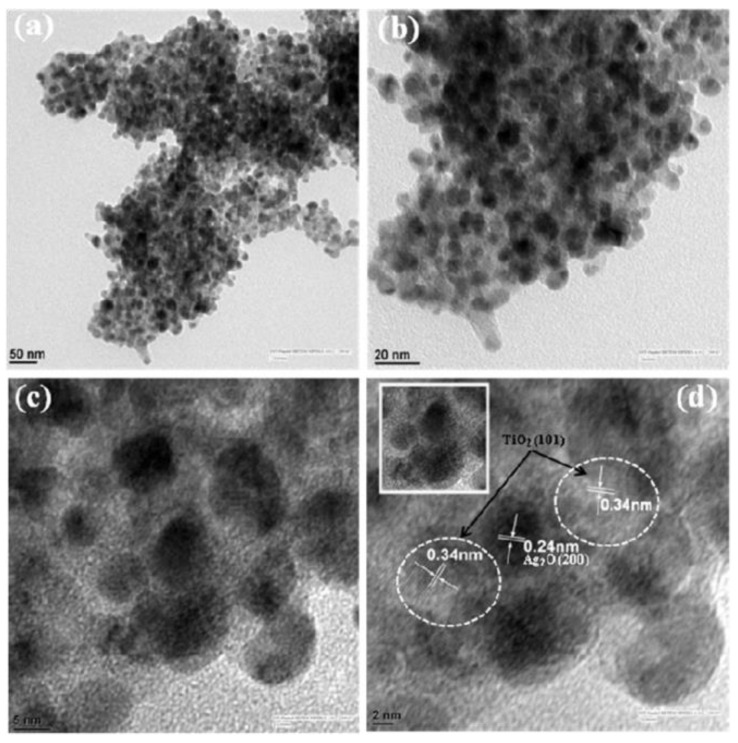
TEM images of Ag_2_O/TiO_2_ quantum dots composite (**a**,**b**) low resolution and (**c**,**d**) high resolution images [123].

The composite based on Ag_2_O/TiO_2_/quantum dots (QDs) with around 10 nm particle size (Figure 16) was synthesized and studied for photocatalytic degradation of levofloxacin. The developed Ag_2_O/TiO_2_ QDs composite showed much higher photocatalytic efficiency (81% in 90 min, pH = 4) as compared to the bare TiO_2_ under visible light illumination (λ > 400 nm). The increased photo-absorption with narrow band gap and reduced electron-hole recombination was attributed to the enhanced photocatalytic activity of Ag_2_O/TiO_2_ QDs [123].

**Figure 17:**
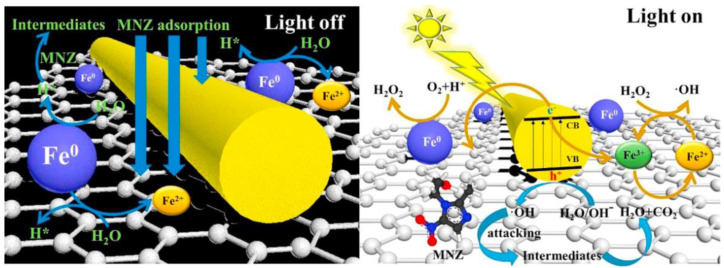
Proposed synergistic photocatalytic mechanism of Fe@GNW nanocomposite for metronidazole degradation [128].

Likewise, the TiO_2_ nanorods modified with Ni(OH)_2_ clusters showed extended adsorption property and improved photocatalytic activity for the degradation of tetracycline under visible light irradiation. In particularly, Ni(OH)_2_ modified coral-like rutile TiO_2_ was found to show ~76% of tetracycline removal efficiency after 2 h, whereas the commercial TiO_2_ (P25) achieved only 57% of removal. In addition, the micro-sized Ni(OH)_2_ modified TiO_2_ composite was found to be easily recovered and also showed significant advantages over nano-sized Ni(OH)_2_ modified TiO_2_ photocatalysts [124]. The fullerene (C_70_) with reduced symmetry structure and larger photo cross-sectional area offered higher electron affinity and effective photo harvesting efficiency. For example, the fullerene incorporated TiO_2_ (C_70_-TiO_2_) hybrid was fabricated and investigated for sulfathiazole degradation and it showed more than 80% degradation efficiency in 90 min under visible light irradiation (300 W xenon lamp, 420 nm cut-off filter). The improved visible photocatalytic performance of C_70_-TiO_2_ was attributed to the hindrance of photogenerated charge carriers recombination and extended visible light adsorption, which received from their strong electron affinity and large photo cross-sectional areas. In addition, the introduction of C_70_ into covalently bonded monolayer TiO_2_ surface was slightly reduced the crystallite size of TiO_2_ and extended their adsorption edge into the visible light region [125]. Similarly, the zeolites are promising carrier material for photocatalytic antibiotics degradation due to their unique porous channel structures, high surface area and excellent adsorption property. For instance, an easily separable zeolite modified-titanium dioxide (TiO_2_/ZEO) composite photocatalyst was successfully synthesized by sol-gel method and showed enhanced catalytic performance for the sulfadiazine degradation. The TiO_2_ nanoparticles around 50 nm sizes were well distributed on zeolite surface, which led to the superior photocatalytic activity and good stability. Compared with bare zeolite, TiO_2_/ZEO composite remarkably improved the photocatalytic efficiency, where more than 90% of sulfadiazine was removed within 120 min, whereas zeolite removed only less than 15% of sulfadiazine. The Ti–O–Si chemical bond formation between TiO_2_ and zeolite was found to be responsible for the observed improved stability of the catalyst. Moreover, the superior adsorption property of zeolite was an important factor for the improved photocatalytic degradation of sulfadiazine. The difference between sulfadiazine adsorption rate and photocatalytic efficiency was also studied [126]. The polyvinyl alcohol and chitosan supported titanium polymer (PVA-CS-TiO_2_) composite as studied for photocatalytic removal of metronidazole in a batch reactor. The PVA-CS-TiO_2_ composite showed the complete removal of metronidazole (100%) within 120 min at a catalyst loading of 0.3 g/L. However, the complete metronidazole removal by TiO_2_ system was observed with 8.3 times higher catalyst dosage under similar conditions [127]. The ternary nanocomposite based on zero-valent iron and graphene-TiO_2_ nanowires (Fe@GNW) was successfully synthesized for the photocatalytic degradation of metronidazole. The synergetic effects of Fe@GNW nanocomposite facilitated the higher separation of photogenerated charge carriers, enhanced surface action, improved adsorption capacity, as well as the magnetic property of the system (Figure 17). As a result, the Fe@GNW nanocomposite displayed superior catalytic activity on the removal of metronidazole (99.3%) as compared to TiO_2_ nanowires (43.0%) and graphene-TiO_2_ nanowires (67.6%). Moreover, the decomposition pathways of metronidazole were proposed based on the observed intermediates [128].

Recently, metal-organic frameworks (MOFs)/semiconductor composite received much attention in antibiotics removal due to their high surface area, porosity, enhanced light absorption and charge transfer. For example, M-MIL-101(Fe)/TiO_2_ composite was successfully synthesized via conventional solvothermal technique and calcination process. The as-developed MOFs-TiO_2_ composite showed excellent photocatalytic degradation efficiency of 92.76% of tetracycline removal within 10 min under solar light (using catalyst 1 g/L, pH = 7 and concentration of tetracycline 20 mg/L), where this outstanding degradation efficiency was significantly higher than that of the recently reported conventional photocatalysts. In the meantime, M-MIL-101(Fe)/TiO_2_ composite was separated easily from the antibiotic solution, where it showed the excellent reusability as well [129]. Similarly, the highly oriented one-dimensional MIL-100(Fe)/TiO_2_ composite nanoarrays revealed the efficient tetracycline degradation of 90.79% in 60 min, which was much higher than that of pristine TiO_2_ nanoarrays (35.22% in 60 min). The incorporation of MIL-100(Fe) MOF was found to potentially improve the photo absorption and also showed the higher electron-hole charge separation. However, the higher percentage of MIL-100(Fe) incorporation was found to limit the photo absorption of TiO_2_ and also affected the overall photocatalytic degradation efficiency [130].

Lately, the designing of larger ZIF-8 particle and TiO_2_ (ZIF-8@TiO_2_) micron composite was found to greatly enhanced the tetracycline adsorption and as well as the photocatalytic degradation efficiency. The ZIF-8@TiO_2_ composite showed the highest rate of degradation k = 0.034 min^−1^, which was about 2.6 times that of ZIF-8 (k = 0.013 min^−1^) and 1.4 times that of pure TiO_2_ (k = 0.034 min^−1^), respectively. The unique porous structure of ZIF-8 and their hybridization with TiO_2_ nanosphere together greatly improved their adsorption capacity. The chemical bonding between ZIF-8 and TiO_2_ offered an ideal way for the photogenerated electron transfer; thereby reduced charge recombination. In addition, ZIF-8@TiO_2_ micron composite also showed the higher surface area and provided more active sites for photocatalytic reaction. Meantime, the ZIF-8@TiO_2_ composite established narrow bandgap energy and facilitated the absorption of visible light photons and potentially improved the photocatalytic performance of the composite [131].

### 3.19. Tungsten Hybrids

The silver (Ag) nanoparticles modified WO_3_ nanoplates showed 96.2% degradation of sulfanilamide under visible light irradiation (200 W Xe arc lamp, specific ranges 420 to 630 nm). This was essential because of the plasmonic properties of silver nanoparticles, which broadened their visible light-absorption and it also acted as electron trapper and thereby enhanced the photocatalytic activity [132]. The homogeneous dispersion of WO_3_ nanoparticle on boron nitride (BN) nanosheets enabled a high surface area and more active sites in the three-dimensional WO_3_/BN nanocomposite. Accordingly, the WO_3_/BN nanocomposite showed the enhanced visible-light photocatalytic degradation of ciprofloxacin (75.0%) the well-dispersed WO_3_ nanoparticles led to the effective interface contact and synergistic effect between WO_3_ and BN, which resulted to the enhanced charge separation for the photocatalytic system [133]. In another study, it was observed that the hybridization of Ag_3_VO_4_ with WO_3_ significantly lower the photocatalytic activity of WO_3_ nanoparticles. Accordingly, the Ag_3_VO_4_/WO_3_ hybrid showed much higher photocatalytic degradation of tetracycline up to 71.2% in 30 min under visible light (300 W xenon lamp, 420 nm cut-off filter), which was around 4.6 times higher than that of pure WO_3_. The formation of heterojunction accelerated the charge carrier separation and as well as prolonged the lifetime of photoexcited charge carriers and thereby enhanced the photocatalytic efficiency. At the meantime, WO_3_ hybridized with Ag_3_VO_4_ could also solve the problem of low photocatalytic activity of WO_3_ [134]. Similarly, the photocatalytic degradation of tetracycline hydrochloride and ceftiofur sodium was demonstrated on the Z-scheme WO_3_/g-C_3_N_4_ hollow microspheres composites (CHMs). Under visible-light irradiation, the WO_3_/g-C_3_N_4_ hollow microspheres composite showed the enhanced photocatalytic efficiency, it degraded around 82% of tetracycline and 70% of ceftiofur sodium within 2 h. The higher photocatalytic activity obtained from the particular structure of WO_3_/g-C_3_N_4_ CHMs. The formed unique hollow microspheres and their cavities together enabled the effective utilization of incident photons to excite the charge carriers and prolong the lifetime of the excited carriers in the system. Therefore, the photoinduced electron-hole pairs were effectively separated and the lifetime of charge carriers reached 2.23 ns, which was obviously extended duration than that of the WO_3_ [135]. On the other hand, the ruthenium (Ru) supported WO_3_/ZrO_2_ composite was developed and it applied for degradation of ampicillin in the presence of UV light. Here, the Ru acted as co-catalyst, which effectively trapped the electrons from WO_3_/ZrO_2_, thereby improved the degradation rate. Accordingly, the Ru/WO_3_/ZrO_2_ found 97% of ampicillin degradation in 180 min, which showed faster degradation rates than WO_3_/ZrO_2_ (96% in 240 min). Moreover, it was found that the ampicillin photocatalytic degradation process was followed by the pseudo-first-order kinetics according to the Langmuir-Hinshelwood model [136].

### 3.20. Zinc Hybrids

**Figure 18:**
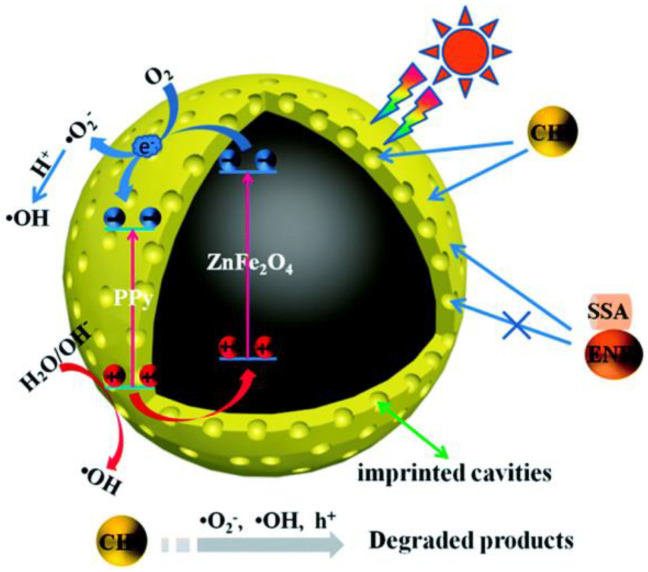
Photocatalytic mechanism of imprinted ZnFe_2_O_4_/PPy composite for selective degradation of ciprofloxacin [142].

The nanoscale magnetic microsphere ZnO-Co_3_O_4_ with well-defined bimetal oxide thin layered structure was developed and it presented excellent oxytetracycline adsorption. The superior adsorption was attributed to its unique structure, high isoelectric point and strong surface complexation. Besides, the novel magnetic microsphere was expected to have the potential application in photodegradation of antibiotics [137]. The vertically aligned ZnO@ZnSnanorod arrays chip was fabricated on Si substrate for the fast degradation of tetracycline hydrochloride in wastewater. It was found that the vertical alignment of the nanorod arrays increased the light-harvesting ability of the system and their polycrystallinity potentially hindered the recombination of photogenerated electron-hole pairs. Accordingly, the ZnO@ZnS nanorods showed efficient photocatalytic degradation of tetracycline up to 80.9% in 140 min under xenon light irradiation (500 W), which also showed excellent recyclability during multiple repeated cycles [138]. Likewise, the uniform distribution of carbon quantum dots (CQDs) on semiconductor surface was found to establish excellent surface contacts and charge transfer. For example, the CQDs (2–4 nm) that well dispersed on ZnS surface was found to show an improved charge separation and higher photocatalytic activity as compared to pure ZnS towards the degradation of ciprofloxacin under simulated solar light (λ > 380 nm) [139]. Similarly, the introduction of carbonaceous materials with metal oxides offered effective surface adsorption properties and improved-photocatalytic degradation efficiency. For instance, 5–10 nm size Ag and ZnO nanoparticles were uniformly deposited on carbonaceous material surface to prepare the Ag/ZnO/C composite photocatalyst. This developed composite exhibited higher adsorption capacity and enhanced UV (95.8% in 35 min) and visible (90.6% in 280 min) driven photocatalytic tetracycline hydrochloride degradation. The synergetic effects between the excellent optical and photophysical properties of the Ag/ZnO/C structure were offered the capability to utilize both of the UV and visible light, efficient photogenerated electron separation and transportation and the increase of the active reaction sites [140]. The RGO-ZnTe hybrid photocatalyst was developed by dispersing the ZnTe nanoparticles on the 2D wrinkled graphene sheet. This as-developed RGO-ZnTe hybrid composite was found to show 2.6 times higher tetracycline degradation efficiency as compared to ZnTe nanoparticles. This observed enhanced visible-light photocatalytic activity was attributed to the synergy effect and strong interaction between the RGO and ZnTe nanoparticles. In addition, the experimental results revealed that the holes played amajor role and superoxide radical minorroleon the tetracycline degradation [141]. The magnetic retrievable imprinted photocatalyst ZnFe_2_O_4_/PPy was designed over the coupling of the imprinted polymer onto ZnFe_2_O_4_ nanocrystals. The imprinted ZnFe_2_O_4_/PPy photocatalyst was allowed to degrade the ciprofloxacin (CIP) and enrofloxacin (ENR) under simulated solar light (200 W tungsten lamp, 320 nm < λ < 780 nm). The obtained results revealed that the imprinted ZnFe_2_O_4_/PPy composites showed higher photocatalytic efficiency and selective degradation of ciprofloxacin and enrofloxacin (CIP-82.76% and ENR-73.78%) as compared to the non-imprinted ZnFe_2_O_4_/PPy composite (CIP-69.89% and ENR-65.34%). Moreover, the degradation efficiency of ciprofloxacin was higher than that of enrofloxacin on the imprinted ZnFe_2_O_4_/PPy composite. This is because of the imprinting cavities that selectively recognized and captured the ciprofloxacin molecules and led photogenerated active free radicals to potentially attack and degrade the ciprofloxacin molecules (Figure 18) [142].

A new attempt of halloysite nanotubes (HNTs) supported ZnO/CeO_2_ heterojunction photocatalyst was fabricated by one step wet-calcination method. The as-fabricated nebula-like ZnO/CeO_2_@HNTs heterostructure composite was found to effectively degrade the tetracycline (94.0% degradation within 60 min) under simulated solar light (300 W Xe lamp equipped with an IR cut filter). The HNTs offered high specific surface area and it also facilitated the well distribution of ZnO and CeO_2_ nanocrystals on HNTs, which ultimately reduced the charge carriers recombination and thereby it increased the photocatalytic efficiency. Moreover, the coexistence of Ce^3+^ and Ce^4+^ states in CeO_2_ has enhanced the electron-hole charge separation through inter-particle charge shifting between CeO_2_ and Ce_2_O_3_ [143]. Similarly, the stable visible light active g-C_3_N_4_-ZnO/HNT nanocomposite effectively degraded tetracycline and showed ~87% removal efficiency in 60 min under visible light irradiation (350 W xenon arc lamp). The HNTs can potentially increase the surface area of g-C_3_N_4_-ZnO, which led to the fast charge transfer and prolonged their lifetime. In addition, as-developed g-C_3_N_4_-ZnO/HNTs composite showed the improved photo-responsive ability in the visible light region and also great stability as compared to the ZnO/HNTs composite [144]. Interestingly, the visible-driven amine-functionalized Al-based porous MOF@Sm_2_O_3_-ZnO nanocomposite (NH_2_-MOF@Sm_2_O_3_-ZnONCP) photocatalyst was studied for the effective degradation of amoxicillin in the presence of ultrasound. As compared to the pure NH_2_-MOF-53(Al) and Sm_2_O_3_-ZnO composites, the NH_2_-MOF@Sm_2_O_3_-ZnO nanocomposite showed excellent photocatalytic activity with around 100% degradation of amoxicillin in 90 min. This enriched activity was attributed to the higher photogenerated charge mobility and extended photo-absorption. In addition, the construction of three-dimensional structure offered the higher structural stability and reusability of NH_2_-MOF@Sm_2_O_3_-ZnO NCPs [145].

## 4. Conclusions

Photocatalytic technique offers a promising solution for the effective degradation of antibiotics in water and wastewater using solar energy. The photocatalytic materials play crucial roles in achieving the complete degradation of these emerging pharmaceutical pollutants. In this direction, the design of hybrid composite photocatalytic materials shows superior catalytic performance as compared to the conventional photocatalysts towards the effective degradation of antibiotics. These hybrid composite photocatalysts overcome the limitation of limited/poor photo-absorption, poor charge separation, slow charge transfer, higher charge recombination, poor surface reaction, lower stability and difficult recovery. In this context, this review sheds insights into the recent progress in the designing, functioning and performance of various hybrid nanocompositephotocatalytic systems for the effective degradation of the antibiotic molecules.
